# Coordination of cortical and thalamic activity during non-REM sleep in humans

**DOI:** 10.1038/ncomms15499

**Published:** 2017-05-25

**Authors:** Rachel A. Mak-McCully, Matthieu Rolland, Anna Sargsyan, Chris Gonzalez, Michel Magnin, Patrick Chauvel, Marc Rey, Hélène Bastuji, Eric Halgren

**Affiliations:** 1 Department of Neurosciences, University of California, San Diego, California 92093, USA; 2 Department of Radiology, University of California, San Diego, California 92093, USA; 3 Central Integration of Pain, Lyon Neuroscience Research Center, INSERM, U1028; CNRS, UMR5292; Université Claude Bernard, Lyon, Bron, France; 4 Aix-Marseille Université, 13385 Marseille, France; 5 INSERM, Institut de Neurosciences des Systèmes UMR 1106, 13005 Marseille, France; 6 APHM (Assistance Publique–Hôpitaux de Marseille), Timone Hospital, 13005 Marseille, France; 7 Unité d'Hypnologie, Service de Neurologie Fonctionnelle et d'Épileptologie, Hôpital Neurologique, Hospices Civils de Lyon, Bron 69002, France; 8 Department of Psychiatry, University of California, San Diego, California 92093, USA

## Abstract

Every night, the human brain produces thousands of downstates and spindles during non-REM sleep. Previous studies indicate that spindles originate thalamically and downstates cortically, loosely grouping spindle occurrence. However, the mechanisms whereby the thalamus and cortex interact in generating these sleep phenomena remain poorly understood. Using bipolar depth recordings, we report here a sequence wherein: (1) convergent cortical downstates lead thalamic downstates; (2) thalamic downstates hyperpolarize thalamic cells, thus triggering spindles; and (3) thalamic spindles are focally projected back to cortex, arriving during the down-to-upstate transition when the cortex replays memories. Thalamic intrinsic currents, therefore, may not be continuously available during non-REM sleep, permitting the cortex to control thalamic spindling by inducing downstates. This archetypical cortico-thalamo-cortical sequence could provide the global physiological context for memory consolidation during non-REM sleep.

## Abstract (web summary)

During non-REM sleep, the thalamus produces spindles and the cortex produces downstates, but the interaction between these two areas in these sleep phenomena is not understood. Here, authors describe the dynamic loop between the thalamus and cortex that organizes the production of spindles and downstates in the human brain.

A fundamental characteristic of mammalian brains is the predominance of ∼0.5–4 Hz slow waves and 0.5–2 s bursts of 10–16 Hz spindles during non-rapid eye movement sleep (NREM) sleep. These rhythms control corticothalamic activity, including cell-firing and plasticity. Slow waves are composed of a downstate (DS), characterized by a period of hyperpolarization, and followed by an upstate, characterized by a period of firing near waking levels^1^. Physiologically, slow wave DSs are more stereotyped in their onset, whereas the upstate exhibits more variability^2^. Spindles are generated in the thalamus by powerful intrinsic H (called the pacemaker current, *I*_h_) and *T* (low-voltage calcium, *I*_T_) currents, and the local circuit between thalamocortical (TC) and thalamic reticular nucleus cells^3^. During NREM sleep, the thalamus is commonly thought to be in a hyperpolarized state that permits activation of *I*_h_, *I*_T_ and rhythmic burst firing, where a burst of action potentials crown a low-threshold calcium spike, allowing spindles to spontaneously emerge^4^.

Although spindles arise primarily thalamically^5^ and slow waves cortically^6^, they each clearly involve both structures^7^,^8^,^9^. Earlier studies described the grouping of spindles by slow waves in the anaesthetized cat cortex and thalamus^10^, in encephalography (EEG) recordings^11^, and in medial depth recordings in humans^12^. This grouping of spindles by slow waves may hierarchically organize memory replay originating in the hippocampus: firing patterns encoding memories are replayed during hippocampal sharp waves and ripples, which are associated with cortical spindles and down-to-upstate transitions in rats^13^,^14^,^15^,^16^. Recently, this hippocampal-cortical coordination was confirmed to underlie memory consolidation^17^. In humans, hippocampal ripples may be grouped by local spindles and slow waves^18^. Behaviourally, memory consolidation is correlated with the coordination of spindles with slow waves in human EEG^19^. Here we demonstrate and quantify the ordered co-occurrence of spindles and DSs in the human cortex and thalamus. Our findings suggest strong functional interactions, enabling a structured neurophysiological environment to emerge during memory replay in humans.

## Results

DSs and spindles were detected on 22 cortical and 8 thalamic, mainly pulvinar, bipolar channels during NREM stages 2 and 3 (N2 and N3) in three patients undergoing evaluation for epilepsy. Using bipolar derivations, where the more lateral contact was subtracted from the more medial adjacent contact along the electrode, ensured that the recorded activity was generated in the local thalamic or cortical grey matter immediately surrounding the contacts. The detected DSs and spindles, therefore, arose in the indicated locations and were not the result of volume conduction^20^. DSs occurred on average more frequently in the cortex (16.3±1.1 min^−1^ per channel) versus thalamus (11.5±2.2, two-way analysis of variance comparing DS densities and spindle densities of the 22 cortical and 8 thalamic channels, main effect of sleep event type, *P*<0.0001), whereas spindles occurred more frequently in the thalamus (7.1±1.8) versus cortex (5±1.3, two-way analysis of variance, *P*<0.0001; Supplementary Table 2). Although the area sampled is limited, these results, together with the earlier latency of DSs in the cortex and of spindles in the thalamus (see ‘Cortical DSs lead thalamic DSs' and ‘Thalamic spindles lead cortical spindles' below) support previous suggestions that the cortex generally initiates DSs and the thalamus initiates spindles.

### Cortical DSs lead thalamic DSs

**Figure 1:**
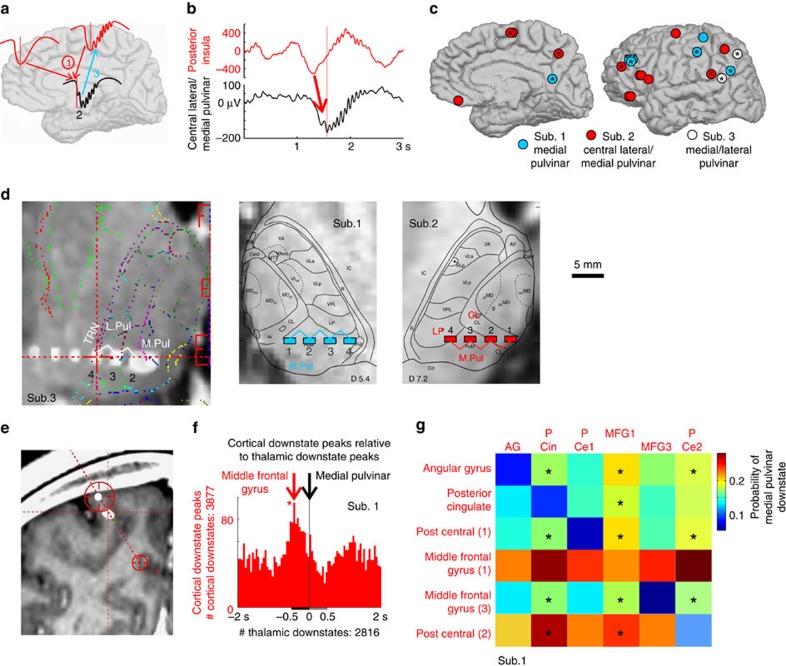
Cortical DSs lead thalamic DSs. (**a**) Overall schema: (1) Cortical DS convergence leads to thalamic DSs; (2) thalamic DSs produce thalamic spindles; and (3) thalamic spindles drive cortical spindles. (**b**) Single trial example of a cortical DS leading a thalamic DS. (**c**) Cortical recording sites. Asterisks indicate cortical locations whose DSs significantly lead DSs in one particular thalamic site, indicated by label, paired to each subject's cortical channels (colour-coded). (**d**) Thalamic recording sites. (**e**) Example transcortical bipolar pair. (**f**) Thalamic DS peaks are locked at 0 ms (vertical black line) and the number of cortical DS peaks occurring within ±2 s are plotted in red 50 ms bins. Cortical DSs occur significantly (*) more often before thalamic DSs than after (Supplementary Table 3A). Representative corticothalamic pair; additional examples in Supplementary Fig. 1A. Similar results were obtained in N2 and N3 (Supplementary Tables 4A & 5A). (**g**) Thalamic DS probability significantly increases when two cortical locations, compared with one, produce a DS. Boxes along the diagonal show the probability of a medial pulvinar DS peak occurring within 500 ms after a cortical DS peak in the cortical channel listed to the left. The remaining boxes indicate the probability of a medial pulvinar DS peak occurring within 500 ms after a cortical DS peak in the cortical channel listed to the left, given a DS in the cortical channel listed on top also occurs within 500 ms after the cortical channel listed to the left (*P<0.05, *χ*^2^, Bonferroni corrected).

Cortical DS peaks significantly led thalamic DS peaks in simultaneously recorded corticothalamic pairs (Fig. 1a–c,f and Supplementary Fig. 1A). Fifty per cent of the 64 pairs exhibited a significant temporal order; of these, ∼90% showed a significant temporal order from the cortex to the thalamus (binomial tests significant at *P*<.05 after Bonferroni correction; see Supplementary Table 3A for all significant pairs and *P*-values). Critically, a thalamic DS was more likely to occur if the number of cortical channels participating in the DS was two (Fig. 1g and Supplementary Note 1) or more (Supplementary Note 1 and Supplementary Fig. 2), compared with when a single channel participated. Owing to the cortex's depolarizing influence on thalamic neurons, cortical DSs, in particular those involving multiple locations, would disfacilitate thalamic neurons (that is, remove the depolarizing drive from the cortex) and may thereby induce thalamic DSs. Cortical DSs in humans are highly variable in their location, extent and synchronization^20^. In significant cortical-to-thalamic pairs, the average delay from cortical DS peak to thalamic DS peak was 218±66 ms, presumably reflecting the integration time needed for the disfacilitating influences of the temporally dispersed cortical DSs to summate on their thalamic targets.

### Thalamic DSs control thalamic spindle onset

**Figure 2:**
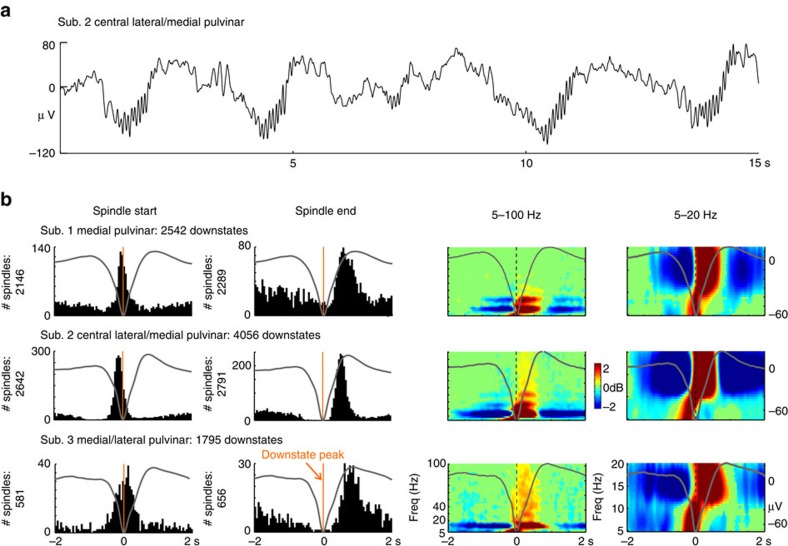
Thalamic DSs trigger thalamic spindles. Spindles start, on average, at the thalamic DS peak and end near the following upstate peak. (**a**) Representative single trace. (**b**) Histograms of thalamic spindle onsets (first column) or spindle terminations (second column) in relation to the thalamic DS peak at 0 ms (vertical orange line) for each thalamic channel; counts in 50 ms bins. Waveforms show the averaged local field potential in each channel. Spectral power from 5 to 100 Hz (third column), or from 5 to 20 Hz (fourth column), averaged on the DS peaks at 0 ms, baseline corrected over entire epoch, thresholded at *P*<0.01, uncorrected. Representative channels; additional channels in Supplementary Fig. 3. Similar results were obtained in N2 and N3 (Supplementary Figs 5–7) and with stricter DS detection parameters (Supplementary Fig. 10).

Given current models of thalamic spindle generation, we were surprised to observe that thalamic DSs tightly controlled thalamic spindling (Fig. 2 and Supplementary Fig. 3). During NREM sleep, the thalamus operates in a burst firing mode governed by the interaction of thalamic *I*_h_ and *I*_T_^3^. Adequate hyperpolarization is required to de-inactivate *I*_T_, meaning to remove *I*_T_ inactivation, and to activate *I*_h_. Subsequent *I*_T_ activation leads to a low-threshold calcium spike crowned by action potentials (that is, burst firing). As a population, this burst firing in TC and thalamic reticular neurons generates the 10–16 Hz oscillations underlying spindles. Consequently, the hyperpolarized state of the thalamus during NREM sleep should spontaneously generate spindles, regardless of the presence or absence of DSs. In contrast, we find that the thalamic DS tightly controls spindle onset in all eight thalamic channels (Fig. 2b and Supplementary Fig. 3, left columns). The normalized proportion of spindles occurring with DSs in the thalamus (0.63±0.21) is nearly double that in the cortex (0.38±0.07, *P*=0.005, one-tailed two-sample *t*-test, unequal variance, 8 thalamic channels and 22 cortical channels), as is the enrichment factor of spindle density around DSs (5.62±2.13 in the thalamus versus 3.15±0.9 in the cortex, *P*=0.007, one-tailed two-sample *t*-test, unequal variance, 8 thalamic channels and 22 cortical channels) (Supplementary Table 6).

We interpret these results as indicating that TC cells are not sufficiently hyperpolarized during NREM sleep to de-inactivate *I*_T_ and activate *I*_h_. Rather, they require the additional hyperpolarization produced by the DS. A corollary of this view is that spindles terminate when the thalamus returns to its baseline NREM membrane potential at the end of the DS. Indeed, plots of thalamic spindle termination versus DSs support this effect (Fig. 2b and Supplementary Fig. 3, second columns). Conversely, the start of the spindle appears to begin the transition from the down-to-upstate, as would be expected from the strong depolarizing currents, which spindles engage. As thalamic DSs appear to be induced by cortical DSs, and thalamic DSs are tightly coupled to the production of thalamic spindles, this allows the cortex a degree of control over thalamic spindle occurrence.

### Thalamic spindles lead cortical spindles

**Figure 3:**
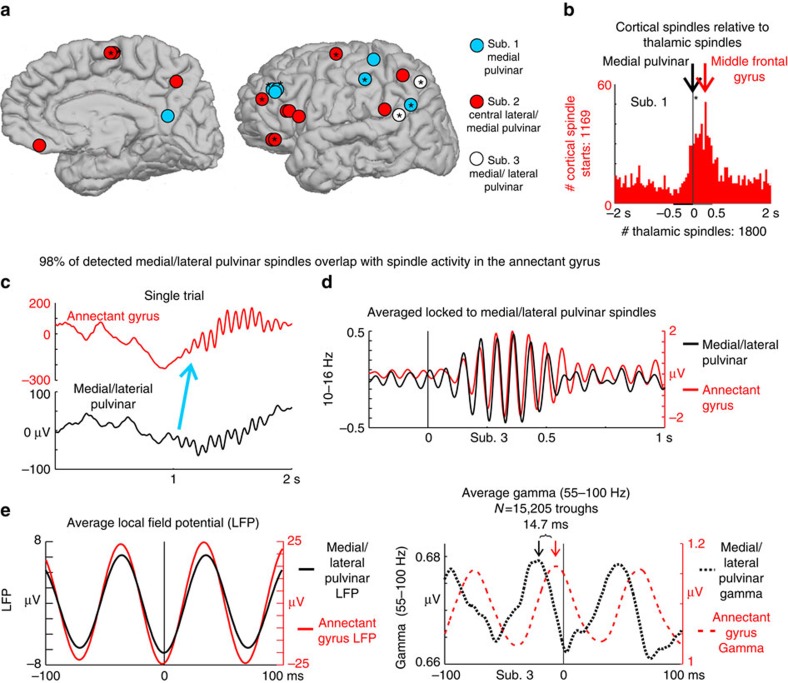
Thalamic spindles lead cortical spindles. (**a**) Cortical sites. Asterisks indicate cortical locations where thalamic spindles significantly lead cortical spindles, in a particular thalamic site/subject. (**b**) Thalamic spindle onsets are locked at 0 ms (vertical black line) and the number of corresponding cortical spindle onsets occurring within ±2 s are plotted in red 50 ms bins. Cortical spindles occur significantly (*) more often after thalamic spindles than before (Supplementary Table 3B). Representative corticothalamic pair; additional examples in Supplementary Fig. 1B. Similar results were obtained in N2 and N3 (Supplementary Tables 4B & 5B). (**c**) Single trial of overlapping medial/lateral pulvinar and annectant gyrus spindles. (**d**) Bandpassed (10–16 Hz) average of the 81% overlapping detected spindles, locked to the pulvinar spindle onsets (vertical black line), indicating synchronous spindle occurrence. (**e**) In the same pair, locked to individual pulvinar spindle troughs (black trace) in the local field potential (LFP), the averaged cortical LFP spindle troughs are synchronous to the pulvinar (left); however, the corresponding high gamma amplitude in the pulvinar leads the cortex by 14.7 ms (right).

In contrast to the consistent lead of cortical DSs over thalamic DSs, thalamic spindle onsets lead those of cortical spindles: 42% of TC pairs showed a significant temporal order, with the thalamus leading in all (Fig. 3a,b, Supplementary Fig. 1B and Supplementary Table 3B). Figure 3c–e demonstrates the focal driving of annectant gyrus spindles by the medial/lateral pulvinar: 98% of detected pulvinar spindles overlapped with detected (81%) or candidate (17%) annectant spindles (Fig. 3c,d; candidate epochs met minimum amplitude-duration criteria, see Methods, ‘Current spindle detection method'). Spindles were considered overlapping between the two channels if any part of a spindle in one channel overlapped at any point in time with a spindle in the second channel. In this pair, high gamma associated with 15,205 spindle troughs in the pulvinar preceded that in the annectant gyrus after ∼15 ms (Fig. 3e, 14.7 ms significantly different than zero; two-tailed paired *t*-test between the time of cortical high gamma amplitude peaks compared with zero and the time of the thalamic high gamma amplitude peaks compared with zero over all subsets of 500 spindle peaks, *P*=0.00035). Overall, these results indicate that thalamic spindles consistently lead cortex spindles, and are consistent with the thalamus focally driving spindle production in certain connected cortical areas (see Supplementary Note 2 and Supplementary Fig. 8 for additional examples of corticothalamic functional connectivity).

### Cortical spindles arrive during down-to-upstate transition

**Figure 4:**
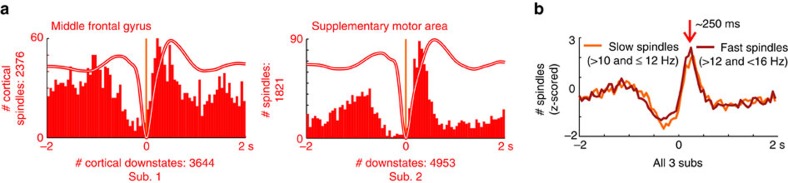
Cortical spindles start during the cortical down-to-upstate transition. (**a**) Histograms of cortical spindle onsets in relation to the cortical DS peak at 0 ms (vertical orange line) for each cortical channel in 50 ms bins. Waveforms show the averaged local field potential in each channel. Representative channels; additional channels in Supplementary Fig. 9. Cortical spindle onset occurs during the down-to-upstate transition in the cortex. Similar results were obtained in N2 and N3 (Supplementary Figs 5–7) and with stricter DS detection parameters (Supplementary Fig. 11). (**b**) Both slow and fast spindles occur on the down-to-upstate transition in the cortex. Results are *z*-scored on 17 channels in which at least 100 slow (>10 and ≤12 Hz) spindles occurred within±500 ms of a DS and on 12 channels in which at least 100 fast (>12 and <16 Hz) spindles occurred within±500 ms of a DS. The red arrow indicates the point, around 250 ms after the cortical DS, when both slow and fast cortical spindles show a peak in start times.

When spindles arrive in the cortex from the thalamus, they arrive during the cortical down-to-upstate transistion (Fig. 4a and Supplementary Fig. 9), with a sharp peak in spindle onset occurring ∼250 ms after the DS peak (Fig. 4b). These findings are consistent across cortical locations (Fig. 4a and Supplementary Fig. 9) as well as for both slow (>10 and ≤12 Hz) and fast (>12 and <16 Hz) spindles (Fig. 4b).

### Proposed model

**Figure 5:**
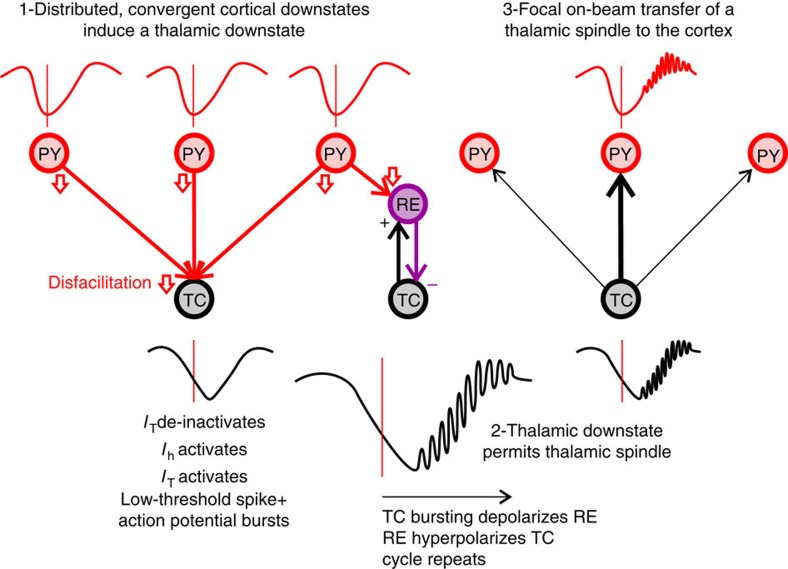
Proposed model for how corticothalamic coordination produces DSs and spindles during NREM sleep. Convergence of cortical DSs produces thalamic hyperpolarization (that is, a DS) by removing depolarizing input from the cortex (that is, disfacilitation, open red arrows; see Fig. 1). This thalamic hyperpolarization de-inactivates *I*_T_ and activates *I*_h_, evoking a thalamic spindle that is reinforced by rhythmic feedback inhibition from thalamic reticular neurons (see Fig. 2). The thalamic spindle, in turn, is focally projected to the cortex (see Fig. 3), arriving during the cortical down-to-upstate transition (see Fig. 4). PY, pyramidal; RE, thalamic reticular; TC, thalamocortical.

Taken together, our results suggest a sequential cortico-thalamo-cortical sequence of DSs and spindles: cortical DSs lead thalamic DSs; the hyperpolarization caused by thalamic DSs triggers thalamic spindle onsets; thalamic spindles lead cortical spindles, driving individual spindle troughs in connected TC pairs; and cortical spindles arrive in the cortex during the cortical down-to-upstate transition (Fig. 5).

## Discussion

During the course of a night's sleep, the human brain produces thousands of DSs and spindles. Yet, we still lack a basic understanding of what triggers these events and how they are coordinated across different brain regions. Here, using rare simultaneous bipolar stereoencephalography (SEEG) recordings in the human thalamus and cortex, we quantify the local generation and interaction of these events between the thalamus and cortex. Although previous studies have observed the grouping of spindles by slow waves, this is the first demonstration and quantification of the temporal order of these sleep events as they occur within and between the human thalamus and cortex. Rather than independent origin of DSs and spindles in the cortex and thalamus, respectively, we find that DSs and spindles occur in a highly regular temporal sequence, both within and between structures. We interpret the implied interactions as arising from the known connections between and within these structures, as well as the powerful voltage-gated channels previously shown in animals to be key for the generation of thalamic spindles.

Our findings appear to be inconsistent with the widespread assumption that the thalamic currents are continuously available during NREM; rather, we propose that these currents become available when DSs hyperpolarize the thalamus, thus allowing spindles to emerge. We show that thalamic DSs systematically follow cortical DSs and are especially likely to be when DSs occur in multiple cortical locations, suggesting that the abrupt silencing of depolarizing projections from the cortex (disfacilitation) may induce thalamic DSs. As thalamic DSs themselves trigger thalamic spindles, this provides a mechanism whereby the cortex may indirectly trigger thalamic spindles. We also show that thalamic spindles begin before cortical spindles, both as the overall spindle envelope and as individual oscillations, consistent with current models wherein thalamic spindles drive cortical spindles. In sum, we interpret our data as indicating that converging cortical DSs induce thalamic DSs, thalamic DSs release thalamic spindles and thalamic spindles drive cortical spindles. Thalamic spindles begin at the peak of the thalamic DS, but as this is delayed from the cortical DS, cortical spindles begin about midway between the down and upstate peaks, when cortical activity networks are re-forming. We propose that this heretofore undescribed interaction between cortex and thalamus can control the initiation and timing of DSs and spindles in order, we hypothesize, to support memory consolidation. There are other interpretations of our data, which we discuss below.

We found that cortical DSs may induce thalamic DSs. In ∼90% of corticothalamic channel pairs with a significant order, the cortical DS led the thalamic DS. Furthermore, DSs occurred ∼50% more often (on a per channel basis) in the cortex than the thalamus. Finally, a thalamic DS was more likely to occur if two or more cortical channels generated a DS, than if only one did. We interpret these observations as being consistent with cortical DSs inducing thalamic DSs via disfacilitation. Cortical pyramidal cells have a relatively high spontaneous firing rate in NREM sleep between DSs, but stop firing (completely or nearly so) during DSs^21^. TC projection cells receive their main excitatory input from the cortex. Removal of this excitatory drive during a cortical DS (that is, disfacilitation) would be expected to hyperpolarize TC cells and thus could induce a DS. It is noteworthy that this disfacilitation would also occur in the nucleus reticularis (RE), whose cells inhibit TC cells. The consequent potential disynaptic disinhibition could be relatively powerful^22^ and perhaps overcome the monosynaptic disfacilitation. In this case, it would be difficult to explain the association of thalamic DSs with cortical DSs, the strong tendency of thalamic DSs to follow cortical DSs, and the accentuation of that tendency when more cortical DSs are present. One possibility that might produce this paradoxical combination if the disynaptic pathway were dominant would be if the cortical projection to RE disrupted ongoing spindle oscillations, which would then decrease TC activity, as we showed in a modelling study^23^. However, this mechanism requires that thalamic spindles precede thalamic DSs, but we found that they follow.

In contrast to this interpretation, we propose that the disynaptic pathway from cortex to TC cells via RE is not immediately engaged by cortical DSs. It is noteworthy that disfacilitation of RE would only have indirect effects on TC if RE were spontaneously active during NREM sleep and limited observations in rats suggest that this is not the case^24^. This interpretation is supported by the lack of high gamma activity in the thalamus before the DS in our data, as evidenced by the lack of a decrease of high gamma during the DS relative to baseline. We conclude that hyperpolarizing DSs in both TC and RE neurons due to converging disfacilitation from cortical DSs is the interpretation most consistent with our findings and the current literature, but this possibility needs to be directly tested.

We also found that thalamic DSs may release thalamic spindles. When thalamic cells are sufficiently hyperpolarized, the *I*_h_ and *I*_T_ currents become available^3^. *I*_h_ slowly depolarizes the cell, until *I*_T_ is opened, causing a burst of action potentials, followed by rebound hyperpolarization due to intrinsic currents and inhibitory feedback from RE. Recovery of *I*_T_, as well as from the after-hyperpolarization due to *I*_h_ and cortical feedback, leads to the next spindle wave. In current theories of thalamic spindle generation^25^, it is assumed that the *I*_h_ and *I*_T_ currents are available quasi-continuously during NREM sleep and so spindles are triggered by small random excitatory fluctuations, resulting in nearly continuous spindling separated by refractory periods. In contrast, based on the very strong association of thalamic spindles with local DSs, with spindle onset times being tightly clustered on the DS peak, we propose that the *I*_h_ and *I*_T_ currents do not become available until the thalamus enters a DS.

One objection to our proposal is that not all thalamic spindles are tightly coupled to thalamic DSs. It is possible that DS-like hyperpolarizing fluctuations, which are not captured by our selection criteria, occur with these non-coupled spindles. This possibility is supported by the similar results we obtained across a twofold range of thalamic DS detection threshold (bottom 40% and bottom 20% of peaks). Extending this view, it is consistent with our data to suppose that the membrane potential of thalamic cells during NREM sleep are close enough to the level where *I*_h_ and *I*_T_ become available that very small fluctuations can release spindles in the absence of a detectable DS. One might expect in this case that the delay from DS onset to spindle onset would be shorter than the ∼500 ms that we observed. However, this delay may be due to the long time required to de-inactivate *I*_T_ and activate *I*_h_ channels, which had been inactivated by depolarization^26^,^27^.

Alternatively, it is possible the non-coupled spindles are generated in another part of the thalamus, where they were coupled to a DS, and then propagated to where we were recording. The limited anatomical sampling is a general constraint on our interpretations. Finally, it is conceivable that *I*_h_ and *I*_T_ are continuously available during NREM sleep and other mechanisms operate to trigger thalamic spindles at the DS peak and to suppress spindles in the periods between DSs. For example, a third structure may project to both the thalamus and the cortex, causing DSs and then spindles. To account for our data, the DS-triggering input would need to be projected to the cortex before the thalamus and the spindle-triggering input would need to be projected to the thalamus before the cortex. Alternatively, some aspect of the down-to-upstate transition may trigger spindles independently in both structures, but at different latencies (immediately in the thalamus and after a 250 ms delay in the cortex). We are not aware of inputs or mechanisms, which satisfy these requirements.

We additionally observed that thalamic spindles may drive cortical spindles. Thalamic and cortical spindles tended to overlap and, in all cases where there was a significant order in their onset, the thalamic spindles led the cortical. Furthermore, when examining those TC pairs that appear to be directly connected, there is a nearly one-to-one overlap between the thalamic and cortical spindles. In such a case, the oscillations in high gamma accompanying the spindles in the thalamus significantly led those in the cortex. As broadband high gamma amplitude is highly correlated with population firing^28^ and as the ∼15 ms delay during spindles from thalamus to cortex is comparable to the TC conduction delay estimated from auditory evoked potentials^29^, these observations are consistent with spindles arising in the thalamus and being projected to the cortex. Furthermore, association of spindles with DSs is much stronger in the thalamus than in the cortex, which is what would be expected if spindles are generated in the thalamus in response to DSs and then are projected to the cortex.

Our interpretation that human sleep spindles are initiated in the thalamus and then projected to the cortex is consistent with the effects of lesions in mammals^5^,^30^. However, an alternative interpretation has been proposed, wherein cortical spindles are gated by the depolarization occurring during the cortical upstate^31^, which is assimilated into a more general tendency of higher frequency oscillations to be gated by lower. Support for this interpretation is the previous observation that the midpoint of human sleep spindles at the scalp or intracranially tends to peak at the midpoint of upstates^11^,^12^. As we are concerned with spindle initiation, we related spindle onset to slow waves in the cortex and found that their peak is about midway between down- and upstate peaks. This is in contrast to the thalamus, where spindle onsets are centered on the DS peaks. It is noteworthy that thalamic DSs peak ∼200 ms after cortical, so cortical spindles occur only shortly after thalamic. Thus, our model provides a consistent mechanistic explanation for the associations and timings of DSs and spindles in thalamus and cortex. However, again, one could posit a third structure, which induces down- and upstates in the cortex and thalamus, as well as initiates spindles, with the requisite timing and correlations.

The recordings and analyses presented here have a number of limitations. Recordings in the thalamus were clearly different than those in the cortex: the thalamic recordings were much lower in amplitude, had small gamma fluctuations and were largely dominated by clear spindles, often occurring in tandem with slow waves. Identification of thalamic DSs was also not as straightforward as in the cortex, due to the difficulty of identifying polarity in relation to high gamma changes (see Methods for details). The thresholds used in previous detection methods for cortical slow waves and spindles did not adequately recognize the discrete events seen in the raw thalamic data. To address this issue, we took the bottom 40% of slow waves as DSs compared with the 20% used in previous methods^12^,^32^. However, when we reanalyse our results using only the bottom 20% of DSs, our results still show the same TC relationships. Our spindle detection method, detailed in the Methods section, optimized spindle detection on a per channel basis, outperforming the previous standard method. Performance of the methods was validated by extensive visual inspection of individual channels and traces.

In addition, the time between zero crossings used for DS detection (0.25–3 s) means that the DSs analysed here were between ∼0.16 and 2 Hz. Studies in anaesthetized cats suggests a difference between slow oscillations (<1 Hz) and delta waves (1–4 Hz)^6^; however, human laminar recordings indicate that the local field potential gradient, multi-unit activity and current source density (CSD) profile are similar from 0.6 to 2 Hz^21^.

Simultaneous recordings in the human thalamus and cortex are extremely rare and only occur in patients with a clinical necessity for such recordings. In the work presented here, all implanted subjects were patients with pharmaco-resistant epilepsy. To exclude as much as possible the influence of epilepsy on our analyses, our inclusion criteria were extremely conservative. Patients and channels were excluded if there was evidence of extensive epileptic activity (see Methods). Time periods in which brief epileptic activity was suspected were also excluded from analysis. Furthermore, the patients studied here presented with temporal lobe epilepsy and seizures related to this type of epilepsy tend to occur during wakefulness rather than sleep^33^. Finally, also due to the limited nature of these recordings, our sample size is limited both in terms of number of subjects, as well as number of sites. Our cortical coverage is small and our thalamic leads are largely confined to the pulvinar. However, given this small sample size, it is still remarkable to find highly consistent temporal relationships between cortical and thalamic spindles and DSs. This implies a global structure of cortico-thalamo-cortical interactions that holds across the cortical mantle.

Studies in humans strongly suggest that memory consolidation occurs during sleep, because manipulating the amount of graphoelements produced during sleep can enhance performance: for example, inducing slow oscillations via transcranial stimulation^34^ or increasing spindle density with zolpidem^35^ improves declarative memory; recently, memory consolidation in humans was found to be highly correlated with the occurrence of spindles at down-to-upstate transitions detected in scalp EEG^19^. Furthermore, this effect may occur very locally^36^.

Hippocampal-dependent declarative memories are thought to be replayed during sleep, to move the memory to the cortex for long-term storage^37^. Hippocampal ripples produced in CA1 and sharp waves generated in CA3 are thought to underlie this memory replay process. In rats, K-complexes have been shown to group these replay periods^38^ and K-complexes, DSs and high voltage spikes have also been correlated with replay^15^. Ripples have also been correlated with cortical slow activity and spindles^39^, and sharp waves are thought to be grouped by the cortical slow wave^13^. Using stimulation, hippocampal-cortical coordination via sharp wave ripples, delta oscillations and ripples, has been demonstrated to underlie memory consolidation^17^. Given the strong anatomical projections from the thalamus to the hippocampus, it is possible that thalamic spindles also drive hippocampal spindles, in parallel to the TC driving demonstrated here, but direct evidence is lacking.

In summary, our results reveal a dynamic cortico-thalamo-cortical loop temporally coordinating DSs and spindles during human NREM sleep. The cortex is silent at the beginning of this transition and achieves waking firing levels at its end^40^. Our results suggest a mechanism whereby the cortico-thalamo-cortical connections and intrinsic thalamic channel properties interact, to ensure that spindles arrive at the cortex when this cortico-cortical associative network is being constructed. We hypothesize that this loop may help organize memory replay.

## Methods

### Recordings

SEEG was performed on 16 patients with pharmaco-resistant epilepsy at Neurological Hospital, Lyon, France or La Timone Hospital, Marseille, France; however, the recordings of 13 patients were excluded, whereas the recordings of 3 patients (2 women, 1 man, age: 40.7±8.1) were included for further analysis (Table 1). The recordings of the 13 patients were excluded for the following reasons: the thalamic leads were implicated in the subject's epilepsy^1^; the patient had already had tissue resected^1^; the thalamic leads were contaminated by artefact^2^, there was breakthrough REM activity in the thalamus^2^; or there were no useable cortical leads^7^.

Fully informed consent was obtained at both hospitals before surgery. The ethics committee of Comité Consultatifs de Protection des Personnes se Prêtant à des Recherches Biomédicales Lyon-Centre Léon Bérard and the Institutional Review Board of the French Institute of Health approved these studies. SEEG recording electrodes were implanted according to the stereotactic technique of 41, to define the epileptogenic area for possible resection. The thalamus was a target of implantation due to its potential role in epileptic discharges. An electrode sampling thalamic areas also had contacts sampling temporal neocortical sites, therefore not increasing patient risk by requiring a separate electrode track.

At both hospitals, each electrode had either 10 or 15 contacts. Each contact was 2 mm long, with a diameter of 0.8 mm and an inter-contact spacing of 1.5 mm.

At the Neurological Hospital, electrodes were perpendicular to the midsagittal plane. The recordings were sampled at 256 Hz and band pass filtered from 0.33 to 128 Hz. The three-dimensional location of electrode contacts was determined directly from stereotactic teleradiographs without parallax performed within the stereotactic frame^42^. These locations were superimposed onto the preimplantation 3T structural magnetic resonance imaging (3D MPRAGE T1 sequence) after alignment with the skull. The locations of cortical and thalamic contacts were determined by reference to the atlases of refs 43, 44. In Fig. 1d, thalamic nuclei are overlaid on each subject's magnetic resonance imaging based on horizontal sections at the indicated distance dorsal to the intercommissural plane from the atlas of ref. 44 (Subject 1 in blue and Subject 2 in red).

At La Timone Hospital, the recordings were sampled at 1,024 Hz and band pass filtered from 0.16 to 340 Hz. Electrodes were localized by fusing the computed tomography (CT) with electrodes implanted and the MRI (Fig. 1d, Subject 3 is in white).

All analyses were performed on bipolar channels derived from subtracting the lateral lead from the medial lead of two adjacent contacts along an SEEG electrode (Fig. 1e). Bipolar channels included for analysis met both physiological and anatomical criteria as described in ref. 20. Only channels exhibiting both slow waves and spindles were included for analysis. Included channels were not part of the epileptogenic focus and did not show frequent epileptiform activity (spikes) or other evidence of damage (pathological slowing). In addition, times in which brief epileptiform activity occurred were excluded from training data sets and post hoc from any slow wave or spindle detections. A total of 22 cortical and eight thalamic bipolar channels were analysed. Sleep staging was performed using the bipolar contacts by a qualified rater (MR) and all reported analyses were confined to N2 and N3.

### DS detection

Slow waves in cortical and thalamic bipolar channels were detected using a method modified from ref. 45, to accurately and maximally identify slow waves in the thalamus. A zero-phase fourth order Butterworth bandpass filter from 0.1 to 4 Hz was applied to each channel over N2 and N3 periods of sleep that were free of epileptic activity. Consecutive zero crossings occurring within 0.25–3 s were then selected. The amplitude of the peak between these zero crossings was calculated and only the top and bottom 40% of peaks were retained as slow waves. DSs and upstates in the cortex were defined based on changes in high gamma power (60–100 Hz), with DSs associated with a decrease in high gamma power around the DS peak and upstates associated with an increase in high gamma power at the time of the upstate peak. This criterion was confirmed by the fact that the DS is always negative in pial minus white matter *trans*-cortical bipolar derivations^20^,^23^.

For supplemental analyses, stricter DS detection parameters were also implemented, whereby consecutive zero crossings were selected between 0.25 and 1 s, and the bottom 20% of peaks were retained as DSs (Supplementary Figs 10 and 11).

**Figure 6:**
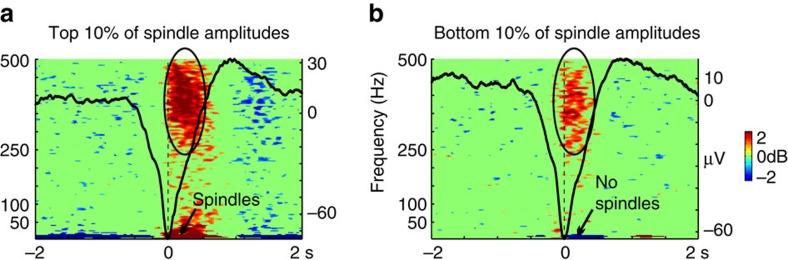
High gamma power changes occur in the thalamus in relation to DSs free of spindles. DSs detected in the medial pulvinar/lateral pulvinar channel of Subject 3 were sorted based on spindle-band amplitudes. The spindle-band amplitude was measured by taking the maximum of the Hilbert amplitude between 0 and 500 ms (DS peak at 0 ms) calculated after applying a 10–16 Hz bandpass filter. (**a**) The DSs corresponding to the top 10% of spindle-band amplitudes show a significant increase in both spindle-band and high gamma power. (**b**) The DSs corresponding to the bottom 10% of spindle-band amplitudes do not show a significant spindle-band increase, but do continue to show a significant increase in high gamma power. For both (**a**,**b**) DSs were epoched from −2.3 to 2.3 s around the DS peaks at 0 ms. Time frequency plots are from 5 to 500 Hz and baseline corrected over the entire epoch. Significance level was set to *P*<0.01, uncorrected. Waveforms show the averaged local field potential for the same trials.

Unlike cortex, thalamic bipolar recordings have no pre-defined consistent relationship between local slow wave generator polarity and anatomical location. Thus, we initially used broadband power increases to tentatively determine the local bipolar polarities corresponding to DSs and upstates. In Subjects 1 and 2, this determination was confounded by the strong effect of spindles, which accounted for most or all of the high gamma increase (Fig. 2 and Supplementary Fig. 3). However, in Subject 3 the wider bandwidth recordings allowed activity in higher frequencies to be measured and this allowed us to confirm the assignment by showing that the high gamma increase was also present in the absence of spindles (Fig. 6). Subject 3 was sampled at 1,024 Hz and therefore could be analysed in the high gamma range above 100 Hz. First, thalamic slow wave peaks were identified in the 0.1–4 Hz bandpassed data. Then the polarity transitions associated with increasing spindling and gamma power were tentatively labelled as down-to-upstate transitions. The peak Hilbert amplitude was calculated on the 10–16 Hz bandpassed signal from 0 to 500 ms after each putative DS peak. The DSs were then sorted based on these peak spindle-band amplitudes. The top 10% of spindle-band amplitudes were strongly associated with high gamma power increases during the transition from the DS to the upstate (Fig. 6a). The bottom 10% did not show any increases in spindle-band power; however, there was still an increase in high gamma power during the transition from the DS to the upstate (Fig. 6b). Is is noteworthy that in this approach, we focused on high gamma increases to define the termination of the DS, rather than the decreases of high gamma as in the cortex. This was necessary because there is very limited baseline high gamma activity in the thalamus, consistent with limited observations in rats^24^.

In addition, we examined high gamma changes as they related to spindle starts and ends in Subject 3, and found that high gamma decreases are associated with the end of spindles rather than DSs (Supplementary Fig. 4). We therefore concluded that our definition of DSs in the thalamus was valid; unlike cortical high gamma, thalamic high gamma increases occur at the transition from DSs to upstates. It is noteworthy that our definition of the thalamic DS, based on high gamma power, is consistent with thalamic and cortical recordings in anaesthetized cats, which found, using local field potential and intracellular recordings, that rebound firing after a DS can occur on the down-to-upstate transition, earlier in the thalamus than the cortex^10^. Recordings in anaesthetized rats also find that in some thalamic nuclei, the slow wave-related firing increases at the down-to-upstate transition^46^. Recordings in anaesthetized mice further show that rebound burst firing in the thalamus occurs during the transition from hyperpolarization^47^.

To use comparable measures across subjects, analyses performed in all three subjects only examine high gamma up to 100 Hz. Only cortical and thalamic DSs were used for further analysis. Post DS detection, artefact rejection was applied to each channel. The difference in consecutive points was calculated to detect any sharp artifacts in the signal and a threshold was set for each channel individually based on visual inspection. In addition, the raw data were epoched±3 s around the DS peaks for each channel. The mean and s.d. were calculated on the absolute value of this raw epoched data. Thresholds for rejection were evaluated visually for each channel. For all but two channels, a threshold of 7 s.d. was applied. For Subject 1, a rejection threshold was set to 10 s.d. for one middle frontal gyrus channel and a rejection threshold was set to 9 s.d. for a second middle frontal gyrus channel. Within channel, if an artefact occurred within an epoch, then that slow wave was rejected.

### Previous spindle detection method

Spindles in cortical and thalamic bipolar channels were detected using a method adapted from that described in ref. 32. The N2 and N3 data for each channel were bandpass filtered using a zero-phase fourth order Butterworth filter from 10 to 16 Hz. The mean of the Hilbert envelope smoothed with a gaussian kernel (300 ms window; 40 ms sigma) was calculated on this band passed signal for each channel. The spindle detection threshold was set at mean +3 s.d., with the start and stop set at mean +1 s.d. We lowered the length required for a spindle from 0.5 to 0.3 s, while maintaining the upper bound of 2 s.

We found, however, that a large number of spindles in the thalamus were not detected using this method. To account for thalamic channels being much smaller in amplitude than cortical channels, and for thalamic channels being largely dominated by spindle activity, we modified the above detection parameters: the spindle detection threshold was lowered to mean +1.5 s.d., while maintaining the mean +1 s.d. for the start and stop for the thalamic channels. Undetected thalamic spindles still remained clearly discernible in the raw data. Therefore, the spindle detection method described below was developed to robustly detect spindles using the same method in both cortical and thalamic channels.

### Current spindle detection method

**Figure 7:**
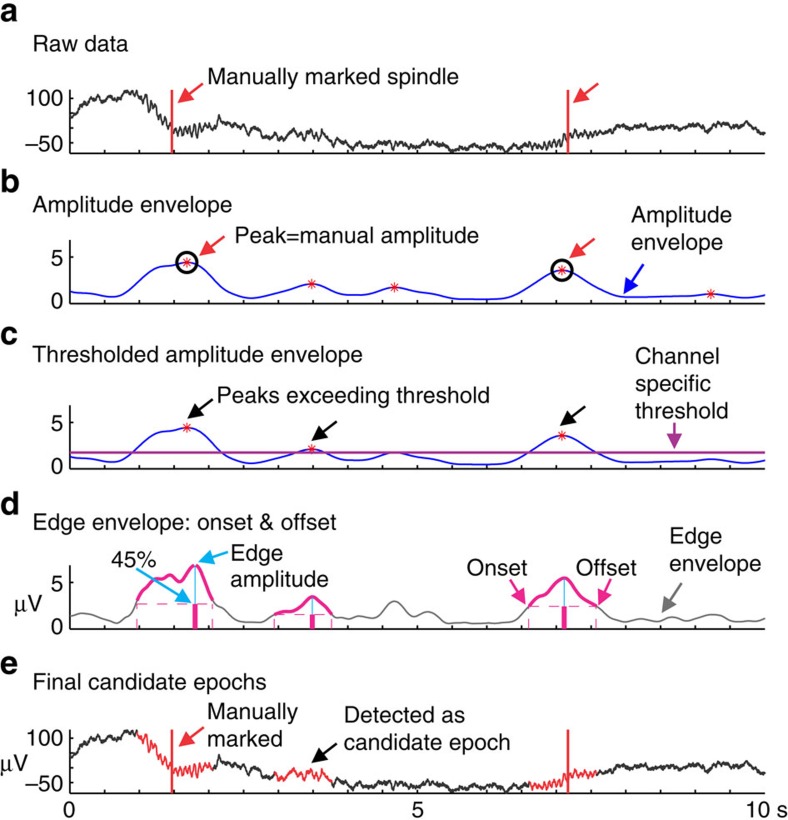
Selection of candidate epochs by current spindle detection method. (**a**) Spindles are manually marked (red lines) on 10 min of N2 and 10 min of N3 raw data, per channel. (**b**) The amplitude of peaks in the amplitude envelope corresponding to manually marked spindles are calculated (manual amplitude, black circles). (**c**) A per channel threshold (purple line) is applied to the amplitude envelope based on the lowest manual amplitude for that channel. Peaks, marked by red asterisks, which exceed that threshold are kept as candidate epochs. (**d**) The closest peak in the edge envelope to the selected peak in the amplitude envelope is detected as the edge amplitude (blue vertical lines). The bounds of the onset and offset of the spindle (pink traces) are calculated at 45% or greater of this edge amplitude. (**e**) The final candidate epochs highlighted in red include epochs where spindles had (red lines) and had not (black arrow) been manually marked.

To begin, individual spindles were exhaustively marked on each channel for 10 min of N2 sleep and 10 min of N3 sleep per subject (marked by R.M.M., Fig. 7a). Spindles over the whole dataset for each subject were then detected in two steps. In the first step, potential spindles are automatically selected; these are the candidate epochs. In the second step, a decision as to whether these candidate epochs are spindles or not was performed using a logistic regression.

In the first step of spindle detection to define the candidate epochs, a 10–16 Hz bandpass was applied to the data and the absolute value of the band passed signal was taken. The resulting signal was convolved with an average Tukey window of 400 ms. This envelope (the edge envelope) was used to define the onset and offset of the candidate epochs (Fig. 7d). A second convolution between the edge envelope and a 600 ms average Tukey window was applied. This envelope (the amplitude envelope) was used to locate the candidate epochs (Fig. 7b).

All peaks in this amplitude envelope where spindles had been manually marked were found (Fig. 7b). These were considered the manual amplitudes. For each channel, the smallest of these manual amplitudes was used as the candidate epoch threshold for that channel. Candidate epochs were therefore located at each peak in the amplitude envelope greater than this smallest manual amplitude for that channel (Fig. 7c). For each of these peaks in the amplitude envelope, the amplitude of the closest peak in the edge envelope was found and considered the edge amplitude. The onset and offset for the candidate epoch was defined as periods where the edge envelope was ≥0.45*the edge amplitude (Fig. 7d). All candidate epochs shorter than 300 ms were discarded. Final candidate epochs included epochs where spindles had and had not been manually marked (Fig. 7e).

In the second step of spindle detection, a set of metrics was computed for each candidate epoch. The spindle amplitude (Amp) was the maximum amplitude of the amplitude envelope within the epoch. Dur was the duration of the spindle equal to onset – offset. The spindle_power was the maximum amplitude of the FFT of the signal between 10 and 16 Hz. The nonspindle_power was the maximum amplitude of the FFT of the signal between 5 and 8 Hz. The F_ratio was the ratio: Spindle_power/Nonspindle_power. The spindle_freq was the frequency where the FFT in the 10–16 Hz range is maximal. The sband_amp was the max of the edge envelope between onset and offset. Similar to the edge envelope, we computed the low band envelope between 4 and 8 Hz, and the high band envelope from 18 to 25 Hz. Lband_amp is the maximum of the low band envelope between onset and offset. Hband_amp is the maximum of the high band power between onset and offset. Stage was the sleep stage. Npeaks was the number of peaks of the spindle. Peakrate is the ratio: npeaks/dur. Peak_intv_mean and peak_intv_std are the peak interval mean and standard deviation. Pr is a binary variable indicating whether peakrate is within spindle range or not.

Using these metrics and the marked periods of data, a logistic regression was used to determine, which candidate epochs were spindles. Spindle events^1^ were all candidate epochs marked as spindles. Non-spindle events (0) were all candidate epochs within the marked periods of time not marked as spindles (Fig. 7e). Because of the observed discrepancies between channels, a model was developed for each channel.

To quantify the correlation between each of our metrics that could cause multicollinearity issues in the stepwise selection, the generalized Variance Inflation Factors (VIFs)^48^ for each predictor in the complete model were computed. As in ref. 48 if the VIF for the predictor with the maximum VIF was >10, this predictor was removed. The step was repeated until no selected predictor had a VIF >10. Once all variables that are not too highly correlated were selected, a bidirectional stepwise selection was applied to select a model^49^ with all the variables that best explain the spindle events. A bootstrap analysis was then performed to ensure that the model selection was stable^50^.

The logistic regression was trained on the marked data and we calculated the probability of each candidate epoch over the whole data set being a spindle. To determine the probability threshold above which a candidate epoch was considered a spindle, the number of false positives (FPs), false negatives (FN), true positives (TP) and true negatives (TN) were computed, for multiple thresholds between 0% and 100% using a leave one out cross validation method. The final threshold was the probability that minimized the difference between the false positive rate (FPR) and the false negative rate (FNR), where FPR=FP/(FP+FN) and FNR=FN/(TP+FN). Analyses were performed using the *glm* procedure, the *vif* procedure of the ‘car' package, the *stepAIC* of the ‘MASS' package and the *boot.stepAIC* procedure of the ‘bootStepAIC' package of the R software^51^.

Periods in which epileptic activity occurred were not used for marking spindles. Spindles that occurred during epileptic times or outside of N2/N3 were eliminated *post hoc*. In addition, *post hoc* artifact rejection as described for slow waves above was applied to spindles, except that the data were epoched from −2 to 4 s around the start of the spindle before calculating the artifact rejection standard deviation threshold and subsequently rejecting epochs.

To determine how well the spindle method described above and the spindle method described in ref. 32 performed, we compared the spindles detected by each method to the manually detected spindles in the 10 min of N2 and 10 min of N3 in each channel (Supplementary Table 1). In applying the method described in ref. 32 to our data, we set the threshold for cortical channels to mean +3 s.d. and the threshold for thalamic channels to mean +1.5 s.d. We also lowered the spindle duration to 0.3 s to match the duration requirements set in our current detection method. For testing of our method, these numbers were calculated using cross-validation. For each method, the number of TPs (manually marked as a spindle, predicted as a spindle), FPs (not manually marked as a spindle, but predicted as a spindle) and FNs (manually marked as a spindle, but not predicted as a spindle) were calculated for each channel and averaged over cortical channels and thalamic channels for each subject.

We found that in this data set our spindle detection method outperformed the traditional detection method outlined in ref. 32, with a greater number of TPs and substantially fewer FNs (Supplementary Table 1). Our spindle detection method also had a lower number of FPs for the thalamic channels in all subjects, but a higher FP rate in the cortex, compared with the previous method. Our hit rate was higher in all cases. Owing to the higher number of FPs in our cortical detections, the false alarm rate was also higher for all subjects in the cortical channels. The *d*' measure indicates how well each detection method discriminated between spindles and non-spindles. The *d*' value was higher in both the cortex and thalamus for all subjects with our current method compared to the previous method. As shown in Supplementary Table 1, *d*' in the cortex was.73 versus 1.82, 1.15 versus 1.85 and 1.25 versus 1.45, comparing the Previous versus Current method in subjects 1, 2 and 3. Similarly, *d*' in the thalamus was 1.05 versus 1.90, 1.52 versus 2.18 and 1.19 versus 1.90, comparing the Previous versus Current method in subjects 1, 2 and 3. *C* measures the bias towards FPs or FNs. In all cases, except for in the cortex for subject 3, the bias was in the same direction for both detection methods. Again, except for in the cortex for subject 3, *C* was smaller for the current detection method compared with the previous method. In sum, these measures indicate that our current detection method discriminated spindles more accurately and with less bias than the previous method, for our data set.

### Analyses

Histograms of the number of events in 50 ms time bins were calculated locking to either DS peaks or spindle starts and examining the timing of DS peaks, spindle starts or spindle ends within or across channels. For example, a histogram height of 10 (on the *y* axis) in the −300 to −350 ms bin (on the *x* axis) in the insula versus pulvinar DS plot would mean that the peaks of ten DSs in the insula channel occurred between 300 and 350 ms before a DS in the indicated pulvinar channel.

Binomial tests of the null hypothesis of random temporal order were performed on the number of DS peaks or spindle starts occurring in one temporal order versus the reverse temporal order for each corticothalamic channel pair over ±500 ms. For slow waves occurring at ∼1 Hz, this time limit was chosen to limit the possibility of false detections (that is, detecting a slow wave from another cycle in the paired channel). Bonferroni correction was applied at *P*<0.05 over all TC pairs, separately for DSs and spindles, which resulted in a significance level of *P*<0.00078.

The average delay from cortical DS peak to thalamic DS peak was calculated on corticothalamic pairs significant for the binomial test described above in the cortical to thalamic direction. For each of these 28 pairs, only DS events in which a cortical DS occurred within 500 ms before a thalamic DS occurred were used. Locking to the times of the thalamic DS peaks for these overlapping events, the average 0.1 to 4 Hz bandpassed cortical DS waveform was calculated. The latency for each corticothalamic pair was then determined by the time of the minimum peak in the cortical DS wave. The overall average latency was calculated by averaging over each of these corticothalamic pairs.

The average delay from DS onset to spindle onset in the thalamus was calculated on the DS waveforms and spindle histograms plotted in Fig. 2b and Supplementary Fig. 3, left columns. DS onset was determined on the averaged 0.1 to 4 Hz bandpassed DS for each thalamic channel. A 20th order polynomial was fit to this average waveform before calculating the polynomial's second derivatives (that is, inflection points). The inflection point closest to the visually determined DS onset was recorded for each thalamic channel. Spindle onset was determined by choosing the latency (middle of the 50 ms bin) corresponding to the spindle peak in each histogram. The average delay for each thalamic channel was calculated by subtracting the spindle onset time from the DS onset.

To determine the relationship of gamma to individual spindle peaks in Fig. 3e, gamma was calculated by taking the analytic amplitude from the Hilbert transform of the data filtered from 55 to 100 Hz using a zero-phase fourth order Butterworth filter. A spline interpolation was applied to the gamma before calculating the time between the gamma peaks in the thalamic and cortical channels. This delay in gamma peak times was used to measure the average delay from thalamic to cortical spindles.

The enrichment factor was calculated to quantify how much more likely an event was to occur in temporal relation with another event. For example, how much more likely spindles were to occur in relation to s (DS), compared with the overall spindle density for a channel. The enrichment factor for each channel was the peak spindle-DS density divided by the overall spindle density. The peak spindle-DS density was calculated per channel by taking the number of spindles in the tallest 50 ms bin within±500 ms of the DS peak. This peak spindle-DS density per minute was computed as follows: (20*60*number of spindles in tallest bin)/number of DSs for that channel. The overall spindle density was calculated by dividing the total number of spindles per channel by the total number of minutes of N2/N3 sleep over which spindles were detected. The enrichment factor was also calculated for DSs in one channel compared to DSs in another channel, spindles in one channel compared with spindles in another channel and spindles in one channel compared with DSs in another channel, as outlined in Supplementary Fig. 8. The calculations were derived in the same way, except that the number of events in the tallest bin would be from one channel and the number of events would be from the other channel.

The proportion of spindles occurring with a DS was calculated to quantify the degree to which spindles are locked to DSs in the thalamus versus the cortex. As cortical spindles arise during the DS-to-upstate transition, whereas thalamic spindles arise during the DS, different time ranges were used for thalamic and cortical channels. For cortical channels, spindles beginning within 750 ms after the DS peak were tabulated, whereas in thalamic channels, spindles were counted if they began within −500 ms to+250 ms of the DS peak. To account for a larger overall number of DSs in the cortex compared with the thalamus, the normalized proportion of spindles occurring with a DS was calculated as follows for each channel: (number of spindles occurring with a DS/total number of spindles)/(total number of DSs/largest total number of DSs within subject).

All analyses were performed in Matlab 2014a (ref. 52). All time frequency plots were generated using EEGLAB^53^.

### Data availability

The data sets analysed during the current study are not publicly available due to privacy concerns as outlined in the consent form but are available from the corresponding author on reasonable request. All analyses were performed using Matlab 2014a and R, along with publically available toolboxes, as detailed in the Methods. Code is available from the corresponding author on reasonable request.

## Additional information

**How to cite this article:** Mak-McCully, R. A. *et al*. Coordination of cortical and thalamic activity during non-REM sleep in humans. *Nat. Commun.* **8,** 15499 doi: 10.1038/ncomms15499 (2017).

**Publisher's note**: Springer Nature remains neutral with regard to jurisdictional claims in published maps and institutional affiliations.

## Supplementary Material

Supplementary Information Supplementary Figures, Supplementary Tables, Supplementary Notes and Supplementary References.

## Figures and Tables

**Table 1:** Patient demographics and clinical information.

**Subject**	**Gender**	**Age**	**Clinical diagnosis**	**Pathological diagnosis**	**Imaging**	**Focus**
Sub1	M	35	Right temporal lobe epilepsy	No pathology obtained	Normal	Temporo-parieto-occipital junction
Sub2	F	37	Temporal lobe epilepsy	No pathology obtained	Normal	Hippocampus
Sub3	F	50	Right temporal occipital epilepsy	No pathology obtained	Normal	Right fusiform gyrus

## References

[b1] Steriade M., Nunez A. & Amzica F. A novel slow (< 1Hz) oscillation of neocortical neurons *in vivo*: depolarizing and hyperpolarizing components. J. Neurosci. 13, 3252–3265 (1993). 8340806 10.1523/JNEUROSCI.13-08-03252.1993 PMC6576541

[b2] Chen J. Y., Chauvette S., Skorheim S., Timofeev I. & Bazhenov M. Interneuron-mediated inhibition synchronizes neuronal activity during slow oscillation. J. Physiol. 590, 3987–4010 (2012). 22641778 10.1113/jphysiol.2012.227462 PMC3476644

[b3] McCormick D. A. & Bal T. Sleep and arousal: thalamocortical mechanisms. Annu. Rev. Neurosci. 20, 185–215 (1997). 9056712 10.1146/annurev.neuro.20.1.185

[b4] McCormick D. A., McGinley M. J. & Salkoff D. B. Brain state dependent activity in the cortex and thalamus. Curr. Opin. Neurobiol. 31, 133–140 (2015). 25460069 10.1016/j.conb.2014.10.003 PMC4375098

[b5] Morison R. S. & Bassett D. L. Electrical activity of the thalamus and basal ganglia in decorticate cats. J. Neurophysiol. 8, 309–314 (1945).

[b6] Steriade M., Nunez A. & Amzica F. Intracellular analysis of relations between the slow (< 1Hz) neocortical oscillation and other sleep rhythms of the electroencephalogram. J. Neurosci. 13, 3266–3283 (1993). 8340807 10.1523/JNEUROSCI.13-08-03266.1993 PMC6576520

[b7] David F. . Essential thalamic contribution to slow waves of natural sleep. J. Neurosci. 33, 19599–19610 (2013). 24336724 10.1523/JNEUROSCI.3169-13.2013 PMC3858629

[b8] Lemieux M., Chen J. Y., Lonjers P., Bazhenov M. & Timofeev I. The impact of cortical deafferentation on the neocortical slow oscillation. J. Neurosci. 34, 5689–5703 (2014). 24741059 10.1523/JNEUROSCI.1156-13.2014 PMC3988418

[b9] Bonjean M. . Corticothalamic feedback controls sleep spindle duration *in vivo*. J. Neurosci. 31, 9124–9134 (2011). 21697364 10.1523/JNEUROSCI.0077-11.2011 PMC3131502

[b10] Contreras D. & Steriade M. Cellular basis of EEG slow rhythms: a study of dynamic corticothalamic relationships. J. Neurosci. 15, 604–622 (1995). 7823167 10.1523/JNEUROSCI.15-01-00604.1995 PMC6578315

[b11] Molle M., Marshall L., Gais S. & Born J. Grouping of spindle activity during slow oscillations in human non-rapid eye movement sleep. J. Neurosci. 22, 10941–10947 (2002). 12486189 10.1523/JNEUROSCI.22-24-10941.2002 PMC6758415

[b12] Andrillon T. . Sleep spindles in humans: insights from intracranial EEG and unit recordings. J. Neurosci. 31, 17821–17834 (2011). 22159098 10.1523/JNEUROSCI.2604-11.2011 PMC3270580

[b13] Battaglia F. P., Sutherland G. R. & McNaughton B. L. Hippocampal sharp wave bursts coincide with neocortical ‘up-state' transitions. Learn. Mem. 11, 697–704 (2004). 15576887 10.1101/lm.73504 PMC534698

[b14] Siapas A. G. & Wilson M. A. Coordinated interactions between hippocampal ripples and cortical spindles during slow-wave sleep. Neuron 21, 1123–1128 (1998). 9856467 10.1016/s0896-6273(00)80629-7

[b15] Johnson L. A., Euston D. R., Tatsuno M. & McNaughton B. L. Stored-trace reactivation in rat prefrontal cortex is correlated with down-to-up state fluctuation density. J. Neurosci. 30, 2650–2661 (2010). 20164349 10.1523/JNEUROSCI.1617-09.2010 PMC2917239

[b16] Molle M., Yeshenko O., Marshall L., Sara S. J. & Born J. Hippocampal sharp wave-ripples linked to slow oscillations in rat slow-wave sleep. J. Neurophysiol. 96, 62–70 (2006). 16611848 10.1152/jn.00014.2006

[b17] Maingret N., Girardeau G., Todorova R., Goutierre M. & Zugaro M. Hippocampo-cortical coupling mediates memory consolidation during sleep. Nat. Neurosci. 19, 959–964 (2016). 27182818 10.1038/nn.4304

[b18] Staresina B. P. . Hierarchical nesting of slow oscillations, spindles and ripples in the human hippocampus during sleep. Nat. Neurosci. 18, 1679–1686 (2015). 26389842 10.1038/nn.4119 PMC4625581

[b19] Niknazar M., Krishnan G. P., Bazhenov M. & Mednick S. C. Coupling of thalamocortical sleep oscillations are important for memory consolidation in humans. PLoS ONE 10, e0144720 (2015). 26671283 10.1371/journal.pone.0144720 PMC4699460

[b20] Mak-McCully R. A. . Distribution, amplitude, incidence, co-occurrence, and propagation of human K-complexes in focal transcortical recordings(1,2,3). eNeuro 2, doi: 10.1523/ENEURO.0028-15.2015 (2015). 10.1523/ENEURO.0028-15.2015 PMC4596022 26465003

[b21] Csercsa R. . Laminar analysis of slow wave activity in humans. Brain 133, 2814–2829 (2010). 20656697 10.1093/brain/awq169 PMC3105490

[b22] Jones E. G. Synchrony in the interconnected circuitry of the thalamus and cerebral cortex. Ann. N Y Acad. Sci. 1157, 10–23 (2009). 19351352 10.1111/j.1749-6632.2009.04534.x

[b23] Mak-McCully R. A. . Synchronization of isolated downstates (K-complexes) may be caused by cortically-induced disruption of thalamic spindling. PLoS Comput. Biol. 10, e1003855 (2014). 25255217 10.1371/journal.pcbi.1003855 PMC4177663

[b24] Bartho P. . Ongoing network state controls the length of sleep spindles via inhibitory activity. Neuron 82, 1367–1379 (2014). 24945776 10.1016/j.neuron.2014.04.046 PMC4064116

[b25] Krishnan G. P. . Cellular and neurochemical basis of sleep stages in the thalamocortical network. Elife 5, e18607 (2016). 27849520 10.7554/eLife.18607 PMC5111887

[b26] Biel M., Wahl-Schott C., Michalakis S. & Zong X. Hyperpolarization-activated cation channels: from genes to function. Physiol. Rev. 89, 847–885 (2009). 19584315 10.1152/physrev.00029.2008

[b27] Lambert R. C., Bessaih T., Crunelli V. & Leresche N. The many faces of T-type calcium channels. Pflugers Arch. 466, 415–423 (2014). 24043572 10.1007/s00424-013-1353-6

[b28] Ray S., Crone N. E., Niebur E., Franaszczuk P. J. & Hsiao S. S. Neural correlates of high-gamma oscillations (60-200Hz) in macaque local field potentials and their potential implications in electrocorticography. J. Neurosci. 28, 11526–11536 (2008). 18987189 10.1523/JNEUROSCI.2848-08.2008 PMC2715840

[b29] Kraus N. & Nicol T. in Encyclopedia of Neuroscience (eds Binder M., Hirokawa N., Windhorst U. Chapter 433, 214–218Springer (2009).

[b30] Timofeev I. & Steriade M. Low-frequency rhythms in the thalamus of intact-cortex and decorticated cats. J. Neurophysiol. 76, 4152–4168 (1996). 8985908 10.1152/jn.1996.76.6.4152

[b31] Steriade M. Grouping of brain rhythms in corticothalamic systems. Neuroscience 137, 1087–1106 (2006). 16343791 10.1016/j.neuroscience.2005.10.029

[b32] Nir Y. . Regional slow waves and spindles in human sleep. Neuron 70, 153–169 (2011). 21482364 10.1016/j.neuron.2011.02.043 PMC3108825

[b33] Crespel A., Coubes P. & Baldy-Moulinier M. Sleep influence on seizures and epilepsy effects on sleep in partial frontal and temporal lobe epilepsies. Clin. Neurophysiol. 111, (Suppl 2): S54–S59 (2000). 10996555 10.1016/s1388-2457(00)00402-8

[b34] Marshall L., Helgadottir H., Molle M. & Born J. Boosting slow oscillations during sleep potentiates memory. Nature 444, 610–613 (2006). 17086200 10.1038/nature05278

[b35] Mednick S. C. . The critical role of sleep spindles in hippocampal-dependent memory: a pharmacology study. J. Neurosci. 33, 4494–4504 (2013). 23467365 10.1523/JNEUROSCI.3127-12.2013 PMC3744388

[b36] Huber R., Ghilardi M. F., Massimini M. & Tononi G. Local sleep and learning. Nature 430, 78–81 (2004). 15184907 10.1038/nature02663

[b37] Rasch B. & Born J. About sleep's role in memory. Physiol. Rev. 93, 681–766 (2013). 23589831 10.1152/physrev.00032.2012 PMC3768102

[b38] Ji D. & Wilson M. A. Coordinated memory replay in the visual cortex and hippocampus during sleep. Nat. Neurosci. 10, 100–107 (2007). 17173043 10.1038/nn1825

[b39] Sirota A., Csicsvari J., Buhl D. & Buzsaki G. Communication between neocortex and hippocampus during sleep in rodents. Proc. Natl Acad. Sci. USA 100, 2065–2069 (2003). 12576550 10.1073/pnas.0437938100 PMC149959

[b40] Steriade M., Timofeev I. & Grenier F. Natural waking and sleep states: a view from inside neocortical neurons. J. Neurophysiol. 85, 1969–1985 (2001). 11353014 10.1152/jn.2001.85.5.1969

[b41] Talairach J. . Surgical therapy for frontal epilepsies. Adv Neurol. 57, 707–732 (1992). 1543089

[b42] Talairach J. & Tournoux P. Co-Planar Stereotaxic Atlas of the Human Brain: 3-Dimensional Proportional System: an Approach to Cerebral Imaging Thieme (1998).

[b43] Duvernoy H. in The Human Brain: surface, Blood Supply, and Three-Dimensional Sectional Anatomy 2nd edn (Springer (1999).

[b44] Morel A., Magnin M. & Jeanmonod D. Multiarchitectonic and stereotactic atlas of the human thalamus. J. Comp. Neurol. 387, 588–630 (1997). 9373015 10.1002/(sici)1096-9861(19971103)387:4<588::aid-cne8>3.0.co;2-z

[b45] Riedner B. A. . Sleep homeostasis and cortical synchronization: III. A high-density EEG study of sleep slow waves in humans. Sleep 30, 1643–1657 (2007). 18246974 10.1093/sleep/30.12.1643 PMC2276133

[b46] Ushimaru M., Ueta Y. & Kawaguchi Y. Differentiated participation of thalamocortical subnetworks in slow/spindle waves and desynchronization. J. Neurosci. 32, 1730–1746 (2012). 22302813 10.1523/JNEUROSCI.4883-11.2012 PMC6703373

[b47] Crunelli V., Lorincz M. L., Errington A. C. & Hughes S. W. Activity of cortical and thalamic neurons during the slow (<1Hz) rhythm in the mouse *in vivo*. Pflugers Arch. 463, 73–88 (2012). 21892727 10.1007/s00424-011-1011-9 PMC3256325

[b48] Kutner M., Nachtsheim C. & Neter J. Applied Linear Regression Models 4th edn McGraw Hill Education (2004).

[b49] Hocking R. R. A biometrics invited paper. the analysis and selection of variables in linear regression. Biometrics 32, 1–49 (1976).

[b50] Austin P. C. & Tu J. V. Bootstrap methods for developing predictive models. Am. Stat. 58, 131–137 (2004).

[b51] R Development Core Team. R Foundation for Statistical Computing R Development Core Team (2004).

[b52] MATLAB, version 8.3.0.532 (R2014a) The MathWorks Inc. (2014).

[b53] Delorme A. & Makeig S. EEGLAB: an open source toolbox for analysis of single-trial EEG dynamics including independent component analysis. J. Neurosci. Methods 134, 9–21 (2004). 15102499 10.1016/j.jneumeth.2003.10.009

