# Supplementary material for: Coordination of cortical and thalamic activity during non-REM sleep in humans
Source: Nat Commun. 2017 May 25;8:15499. doi: 10.1038/ncomms15499 (PMC5458505; doi:10.1038/ncomms15499)
Supplement: Supplementary Information — Supplementary Figures, Supplementary Tables, Supplementary Notes and Supplementary References. [file ncomms15499-s1.pdf]

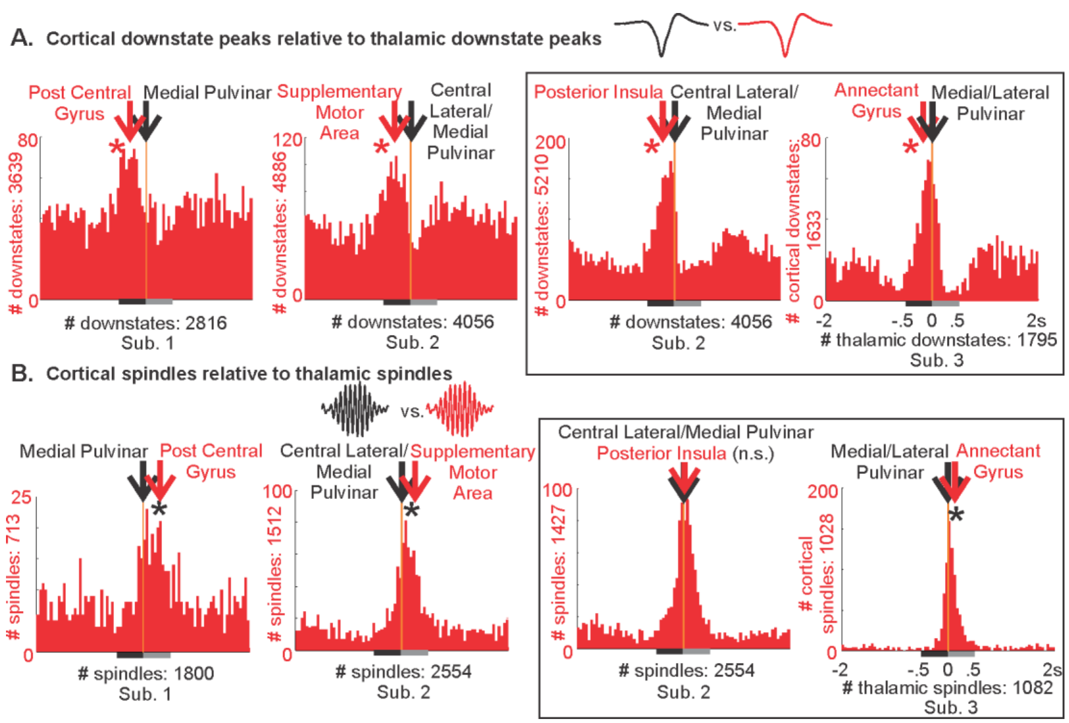

Supplementary Figure 1. Histograms of corticothalamic pairs for downstates and spindles. A. Timing of cortical downstates relative to thalamic downstates: thalamic downstate peaks are locked at 0ms (vertical orange lines) and the corresponding cortical downstate peaks are plotted in red 50ms bins. In all cases, the time of maximal cortical downstate occurrence (red arrow) leads thalamic downstates (black arrow). The number of cortical downstates was significantly more in the 500ms prior to the thalamic downstates, as compared to the 500ms after (*, binomial test, Bonferroni corrected at $p<0.05$; all p-values listed in Supplementary Table 3A). Box indicates relatively strongly connected corticothalamic pairs (see Supplementary Note 2). B. Timing of cortical spindles relative to thalamic spindles: thalamic spindle onsets are locked at 0ms (vertical orange lines) and the corresponding cortical spindle onsets are plotted in red 50ms bins. All pairs are the same as in A. The number of cortical spindles was significantly more in the 500ms after the thalamic spindles, as compared to the 500ms prior (*, binomial test, Bonferroni corrected at $p<0.05$; all p-values listed in Supplementary Table 3B), for all but one pair. In the central lateral/medial pulvinar versus posterior insula (n.s.), the spindles onset nearly simultaneously.

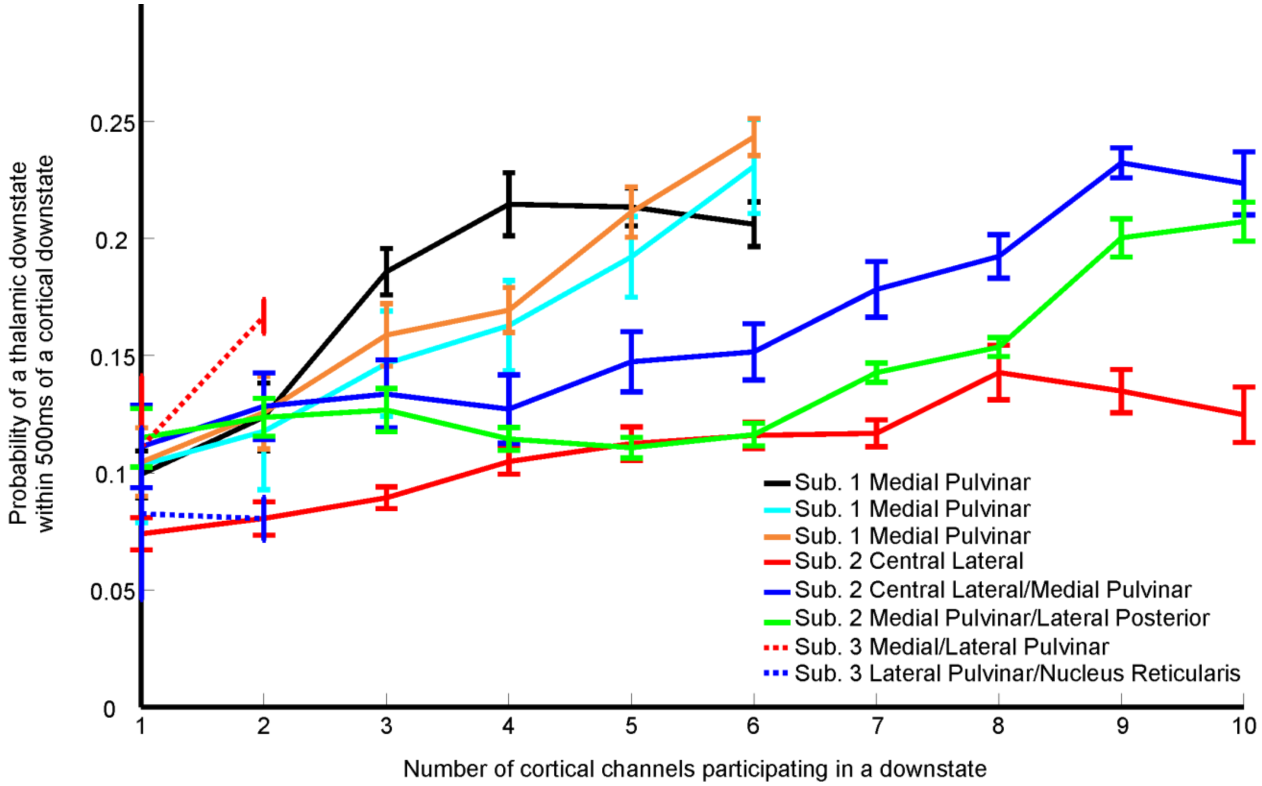

Supplementary Figure 2. The probability of a thalamic downstate increases as the number of cortical channels participating in a cortical downstate increases. For each subject, the number of downstates occurring within 500ms after one other cortical channel, up to the maximum number of cortical channels, was calculated. For each thalamic channel, the probability of thalamic downstates occurring within 500ms after cortical downstates was calculated for all combinations of cortical channels. When a downstate occurred in two or more cortical channels, the downstate peak of the locked cortical channel was used to search for thalamic downstates. Each colored line represents the mean ± SEM for each thalamic channel. In most thalamic channels, as more cortical channels participate in a downstate, the probability of a thalamic downstate increases.

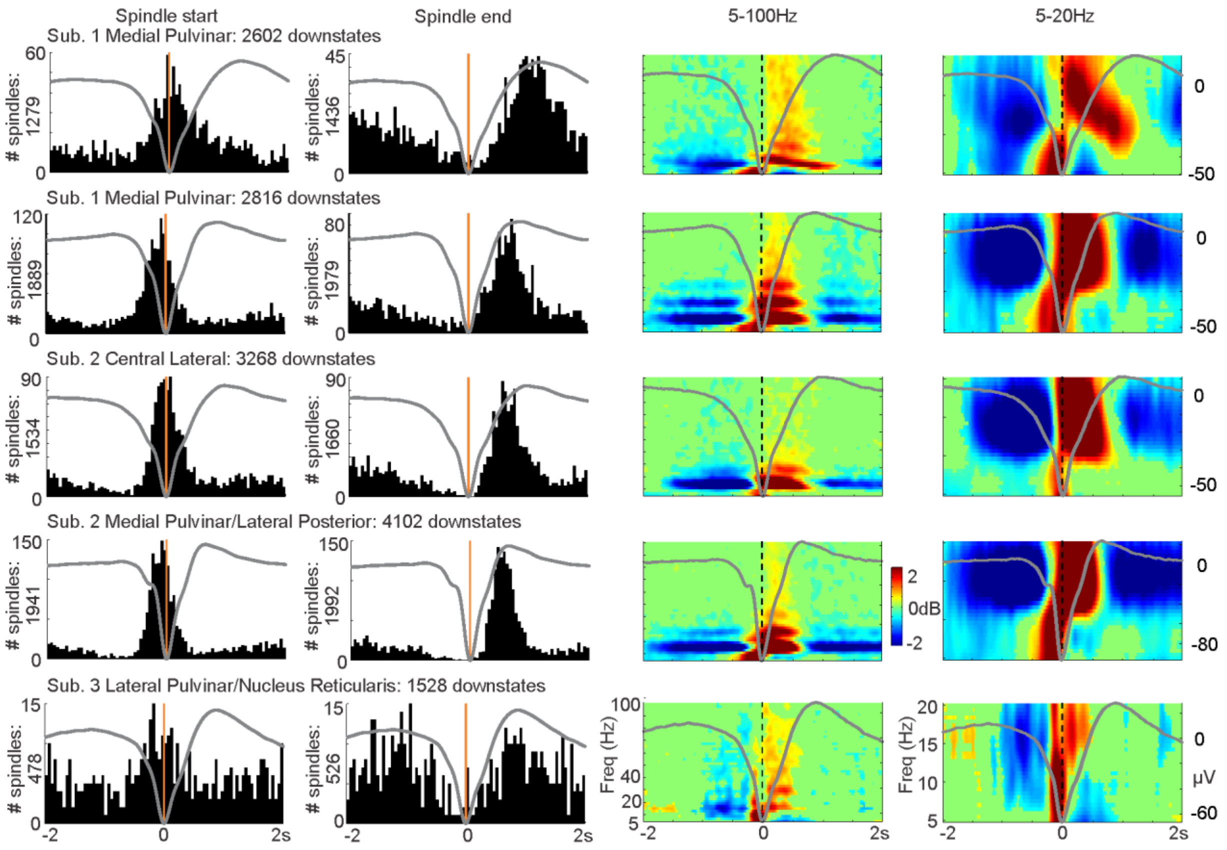

Supplementary Figure 3. On average, spindles start at the thalamic downstate peak, and end at the following thalamic upstate peak. Histograms of thalamic spindle onsets (first column) or spindle terminations (second column) in relation to the thalamic downstate peak at 0ms (orange vertical lines) for each thalamic channel in 50ms bins. Spectral power from 5-100Hz (third column), or from 5-20Hz (fourth column) averaged on the downstate peaks at 0ms, baseline corrected over entire epoch, thresholded at $p<0.01$, uncorrected. Channels plotted here and in Fig. 2B comprise all thalamic channels. Waveforms show the averaged local field potential in each channel.

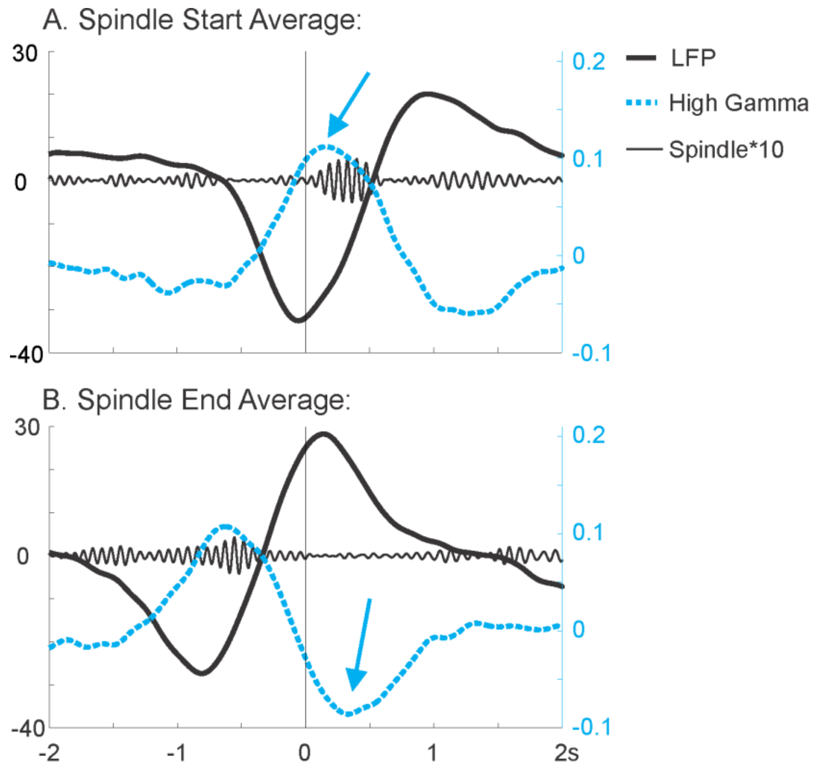

Supplementary Figure 4. High gamma decreases in the thalamus are related to the end of spindles. In Subject 3, the local field potential (0.1 to 4Hz bandpass, thick black line), high gamma amplitude (Hilbert analytic amplitude on data bandpassed from 250 to 500Hz and then bandpassed from 0.1 to 4Hz, blue dotted line), and spindle (10-16Hz bandpass, thin black line) are plotted for the medial pulvinar/lateral pulvinar channel. The spindle trace has been multiplied by 10 for display purposes. Blue arrows indicate the high gamma increases or decreases in each subplot. A. The local field potential (LFP), high gamma amplitude, and spindle locked to the start of spindles. There is a clear high gamma increase after the downstate LFP peak that coincides with the start of spindles. B. The LFP, high gamma amplitude, and spindle locked to the end of spindles. A clear high gamma decrease occurs at the end of spindles.

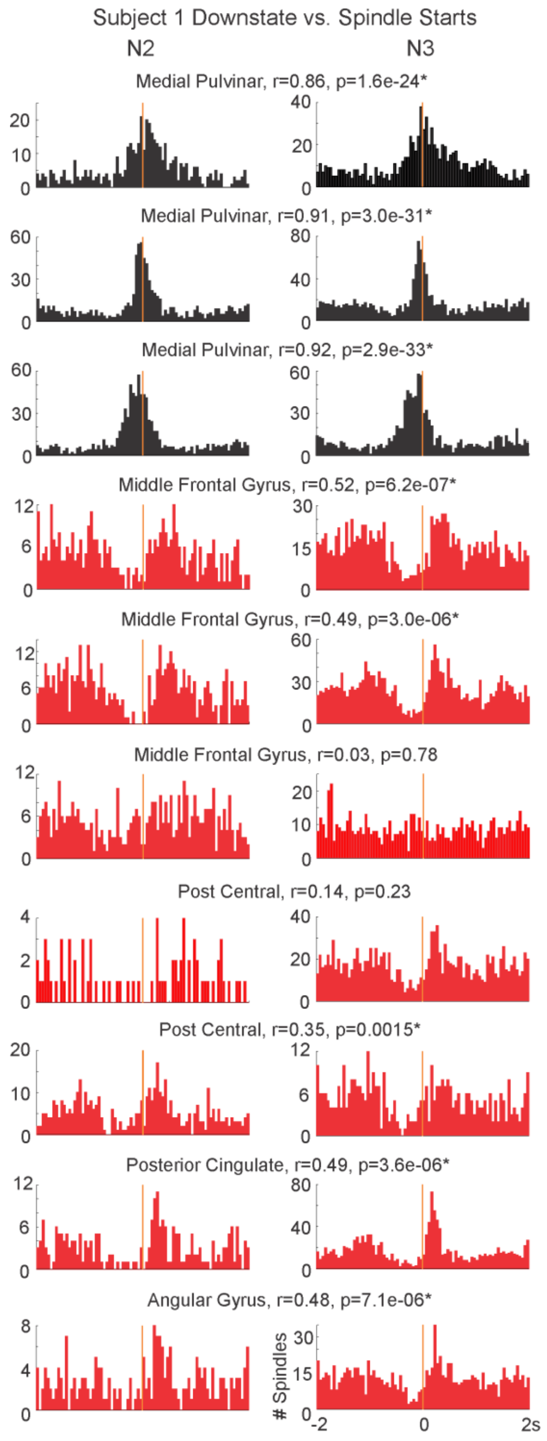

Supplementary Figure 5. Downstate versus spindle relationship in N2 versus N3 for Subject 1. In each row, the spindle starts occurring within ±2 seconds of N2 downstate (left) or N3 downstate (right) peaks are plotted in 50ms bins for each channel. Vertical orange lines mark the local downstate peaks at 0ms for each channel. The overall shape of the spindle distributions locked to downstates appears similar in N2 and N3 for each channel: thalamic spindles occur clustered around the downstate peak (black histograms), while cortical spindles arise after the downstate peak (red histograms). For each channel, the correlation coefficient between N2 and

N3 spindles occurring in relation to downstates was calculated. The location, correlation coefficient, r, and the p-value for the correlation, are calculated for each channel. P-values<0.05, corrected for multiple comparisons, are marked with an asterisk and indicate that the distributions arising in relationship to downstates are significantly correlated between N2 versus N3 spindles. Here, one middle frontal gyrus channel and one post central channel do not have significant correlations; however, the pattern of spindles related to downstates is less clear for this middle frontal gyrus channel than other channels and the number of spindles arising in N2 for this post central channel is small, although they both show a tendency to occur after the downstates.

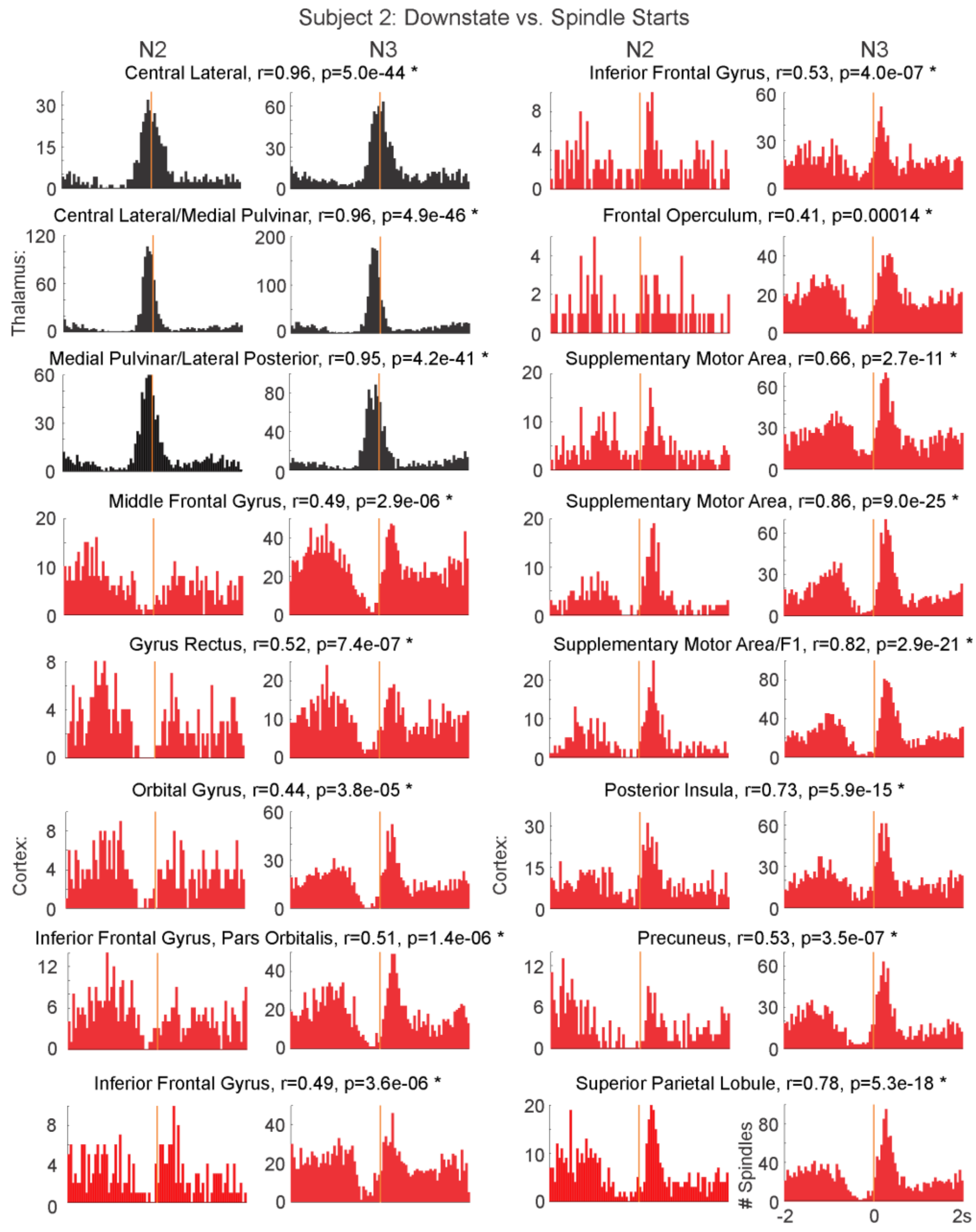

Supplementary Figure 6. **Downstate versus spindle relationship in N2 versus N3 for Subject 2**. The spindle starts occurring within ±2 seconds of N2 downstate (left) or N3 downstate (right) peaks are plotted in 50ms bins for each channel. Vertical orange lines mark the local downstate peaks at 0ms for each channel. The overall shape of the spindle distributions locked to downstates appears similar in N2 and N3 for each channel: thalamic spindles occur clustered around the downstate peak (black histograms), while cortical spindles arise after the downstate peak (red histograms). For each channel, the correlation coefficient between N2 and N3 spindles occurring in relation to downstates was calculated. The location, correlation coefficient, r, and the p-value for the correlation, are calculated for each channel. P-values<0.05, corrected for multiple comparisons, are marked with an asterisk and indicate that the distributions arising in relationship to downstates are significantly correlated between N2 versus N3 spindles. All channels show a significant correlation.

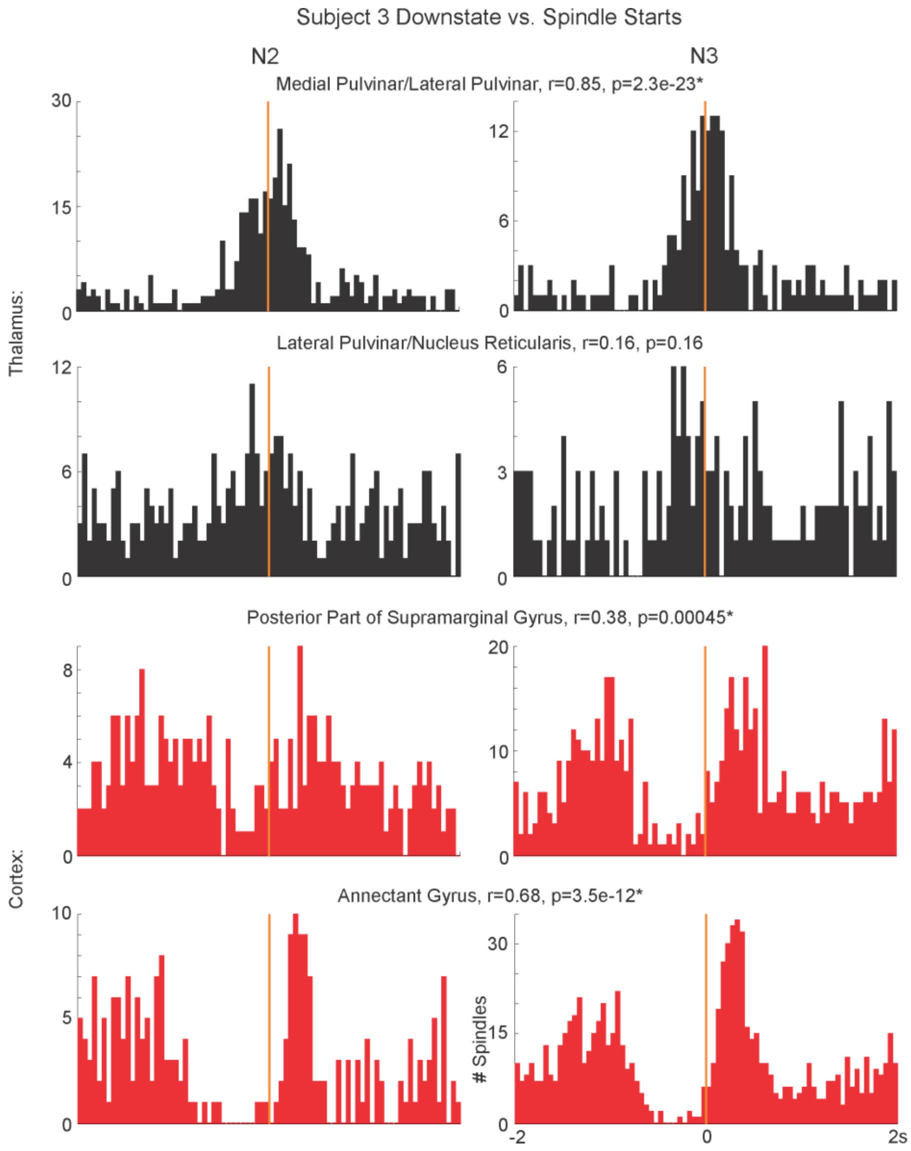

Supplementary Figure 7. **Downstate versus spindle relationship in N2 versus N3 for Subject 3.** The spindle starts occurring within ±2 seconds of N2 downstate (left) or N3 downstate (right) peaks are plotted in 50ms bins for each channel. Vertical orange lines mark the local downstate peaks at 0ms for each channel. The overall shape of the spindle distributions locked to downstates appears similar in N2 and N3 for each channel: thalamic spindles occur clustered around the downstate peak (black histograms), while cortical spindles arise after the downstate peak (red histograms). For each channel, the correlation coefficient between N2 and N3 spindles occurring in relation to downstates was calculated. The location, correlation coefficient, r, and the p-value for the correlation, are calculated for each channel. P-values<0.05, corrected for multiple comparisons, are marked with an asterisk and indicate that the distributions arising in relationship to downstates are significantly correlated between N2 versus N3 spindles. All channels except for the lateral pulvinar/nucleus reticularis have a significant correlation. In this channel, the spindles are still clustered around 0 for N2 and N3, but appear more clustered to the left of 0 in N3.

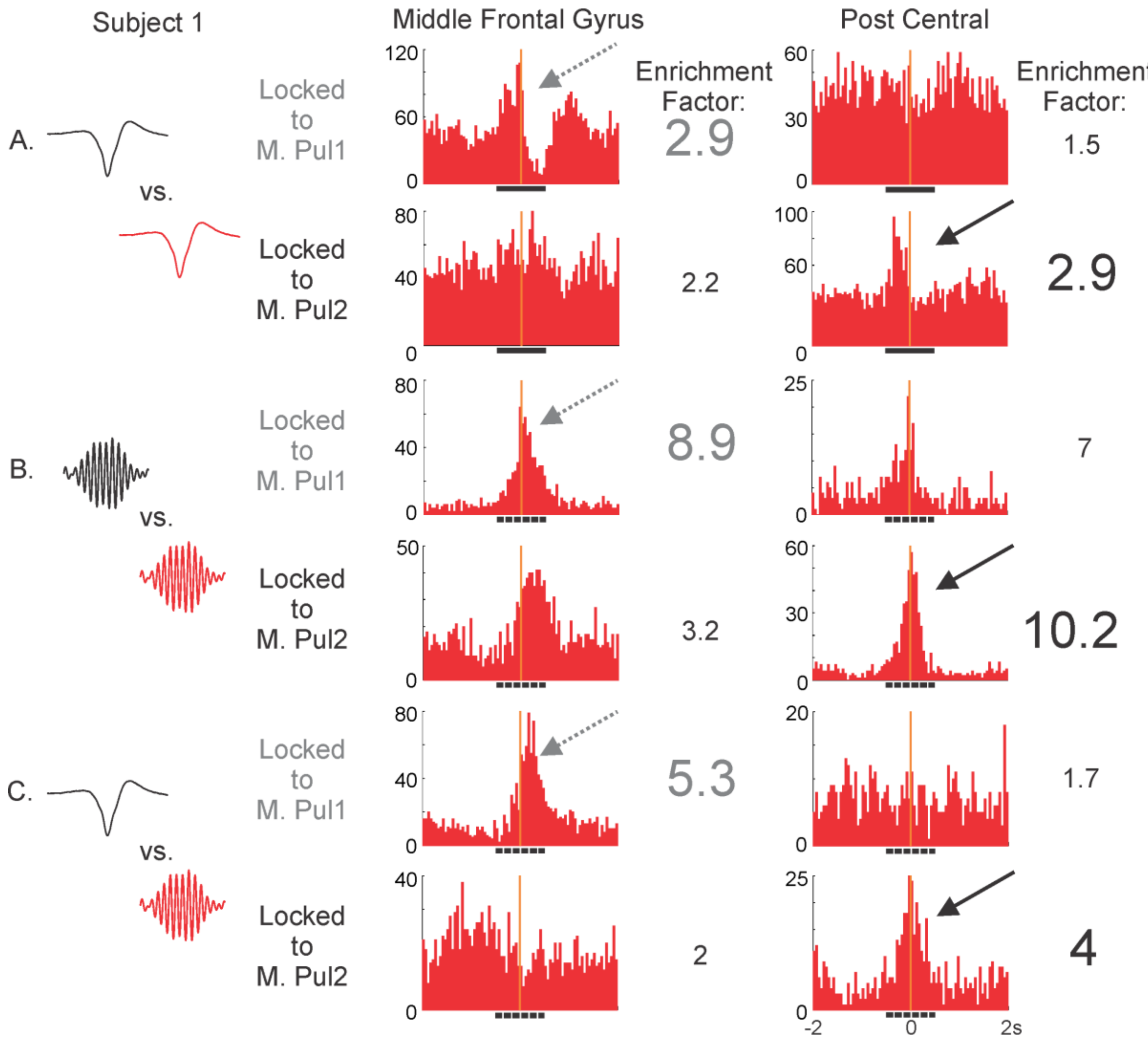

Supplementary Figure 8. Different parts of medial pulvinar are related to different parts of cortex in Subject 1 during downstates and spindles. Events arising in the middle frontal gyrus channel (left column) or the post central channel (right column) are locked to events in the medial pulvinar channels M. Pul1 (labeled in grey) or M. Pul2 (labeled in black). The three event combinations are: downstate versus downstate (A), spindle versus spindle (B), and downstate versus spindle (C). The enrichment factor (see Methods) denotes how the density of downstates or spindles in the cortical channel is increased relative to its normal density when locked to the downstate or spindle events in the medial pulvinar channel. Solid lines (downstates) or dashed lines (spindles) under each histogram denote the ±500ms over which the enrichment factor was calculated. Grey arrows and grey enrichment factors indicate the related M. Pul1/Middle Frontal Gyrus pair. Black arrows and black enrichment factors indicate the related M. Pul2/Post Central pair. A. Downstate versus downstate. Downstates in the Middle Frontal Gyrus peak just before the M. Pul1 downstates at 0ms and drop sharply afterwards. In contrast, Post Central downstates locked to M. Pul1 downstates occur more randomly in time. These patterns reverse when the cortical channels are locked to the second thalamic channel, M. Pul2. The downstate versus downstate enrichment factor is greatest for M. Pul1/ Middle Frontal Gyrus and M. Pul2/ Post Central (2.9 for both), compared to 1.5 for M. Pul1/ Post Central and

2.2 for M. Pul2/ Middle Frontal Gyrus. B. Spindle versus spindle. The highly associated M. Pul1/ Middle Frontal Gyrus and M. Pul2/ Post Central thalamocortical pairings share spindles centered on 0 and both have high enrichment factors (8.9 and 10.2, respectively). In comparison, M. Pul1/ Post Central exhibits a lower degree of association with an enrichment factor of 7 and M. Pul2/ Middle Frontal Gyrus has the lowest spindle enrichment factor at 3.2. M. Pul2 and Middle Frontal Gyrus still appear to be highly related, but have a low enrichment factor, due to the skewing to the right of Middle Frontal Gyrus spindles. C. Downstate versus spindle. The M. Pul1/ Middle Frontal Gyrus pair shows that Middle Frontal Gyrus spindles occur during and just after the M. Pul1 downstate peak, with an enrichment factor of 5.3. Similarly, the Post Central spindles occur during the M. Pul2 downstate peak, with an enrichment factor of 4. Conversely, the opposite pairings show fairly flat histograms with low enrichment factors (2 for M. Pul2/ Middle Frontal Gyrus and 1.7 for M. Pul1/ Post Central). These plots demonstrate a double dissociation wherein one medial pulvinar contact pair is more closely related (in downstates, spindles, and their combination) to the middle frontal gyrus, and the other to the post central gyrus.

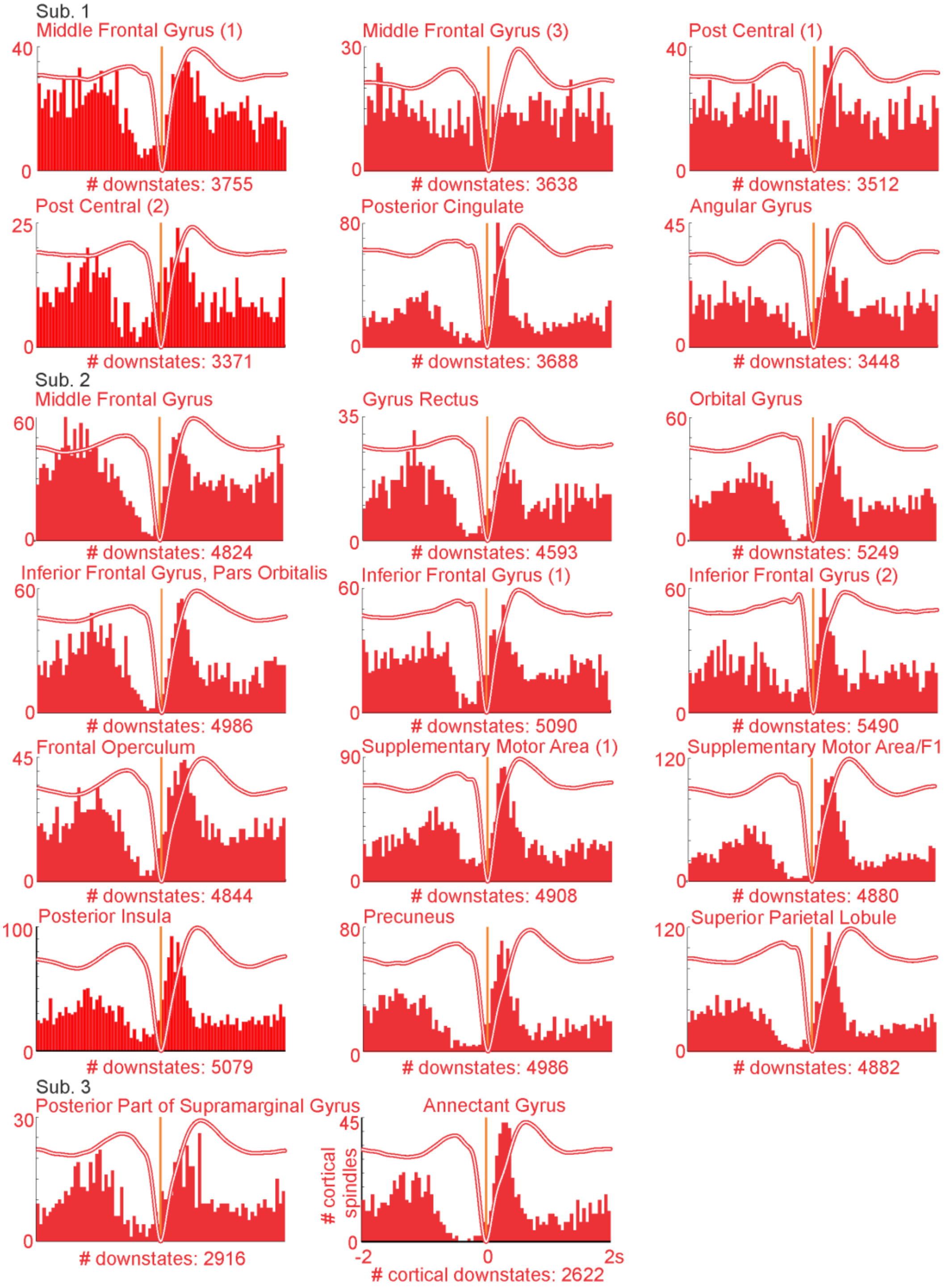

Supplementary Figure 9. Cortical spindles start during the cortical down-to-upstate transition. Cortical downstate peaks are locked at 0ms (vertical orange lines) and the corresponding cortical spindle onsets are plotted in red 50ms bins for each channel. Channels plotted here and in Fig. 4 comprise all cortical channels. Waveforms show the averaged local field potential in each channel.

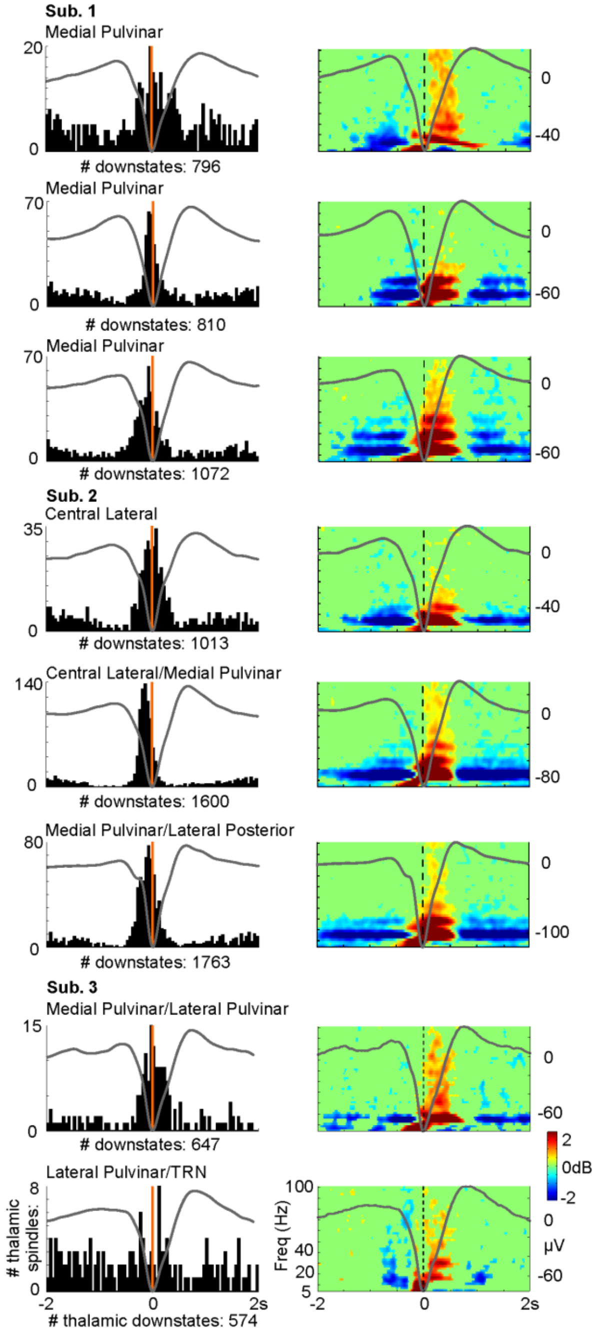

Supplementary Figure 10. Thalamic spindles still start at the thalamic downstate peak when stricter thalamic downstate detection parameters are applied. When downstate detection parameters are changed to only select the bottom 20% of downstate peaks whose zero crossings occur between 0.25 to 1secs, the thalamic spindles still start during the thalamic downstate. Histograms of thalamic spindle onsets (left column) in relation to the thalamic downstate peak at 0ms (vertical orange lines) for each thalamic channel in 50ms bins. Spectral power from 5-100Hz (right column), averaged on the downstate peaks at 0ms, baseline corrected over entire epoch, thresholded at $p<0.01$, uncorrected. All 8 thalamic channels are plotted. Waveforms show the averaged local field potential in each channel.

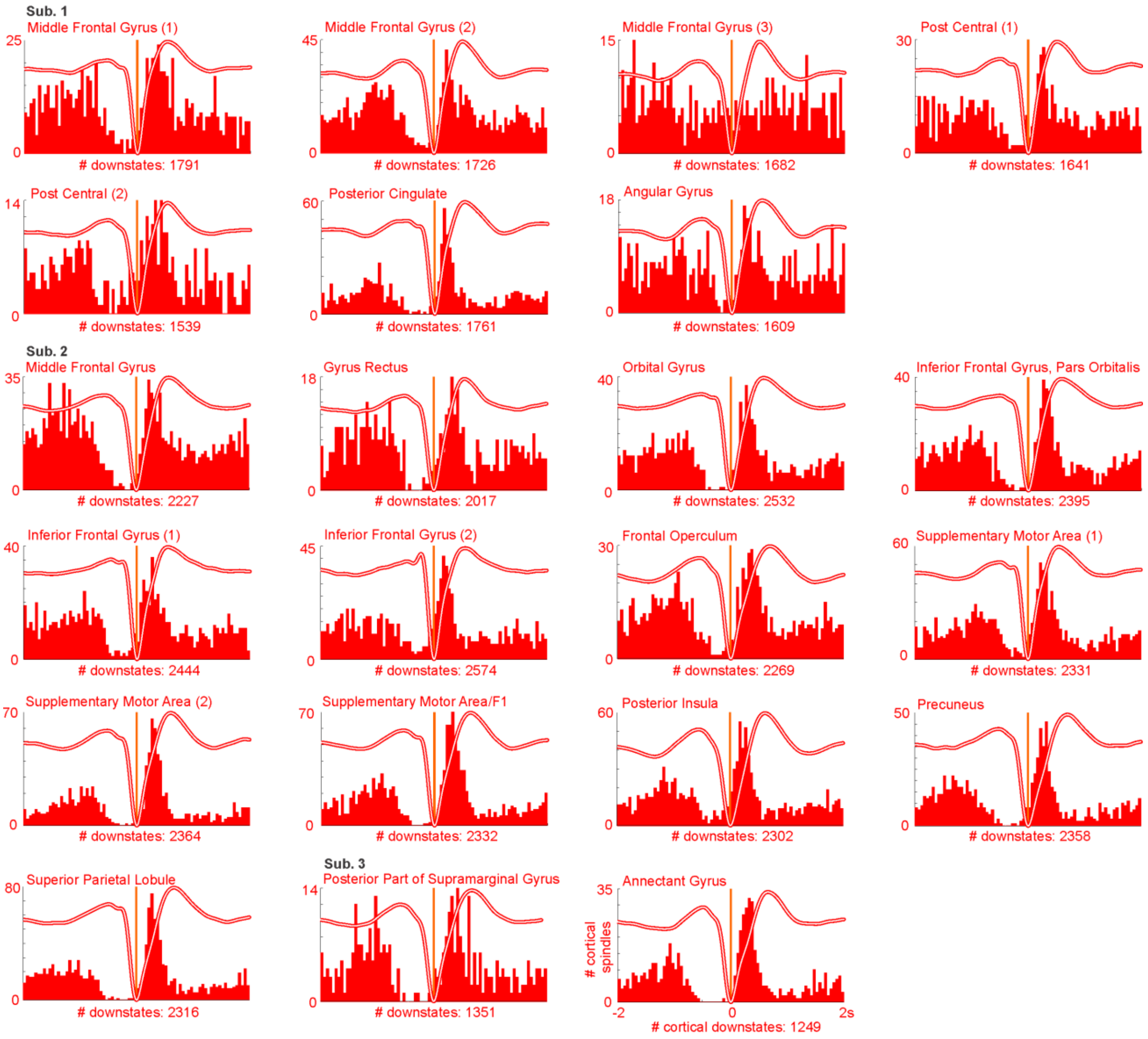

Supplementary Figure 11. Cortical spindles still start during the cortical down-to-upstate transition when stricter cortical downstate detection parameters are applied. When downstate detection parameters are changed to only select the bottom 20% of downstate peaks whose zero crossings occur between 0.25 to 1secs, the cortical spindles still start during the cortical down-to-upstate transition. Cortical downstate peaks are locked at 0ms (vertical orange lines) and the corresponding cortical spindle onsets are plotted in red 50ms bins for each channel. Waveforms show the averaged local field potential in each channel. All 22 cortical channels are plotted.

Subject 1	True Negative	False Negative	False Positive	True Positive	Hit Rate	False Alarm Rate	d'	C
**Cortex**								
Previous	1673	391	357	283	0.419881	0.175862	0.729	0.567
Current	1718	112	375	499	0.816694	0.179169	1.821	0.008
**Thalamus**								
Previous	303	166	151	451	0.730956	0.332599	1.048	-0.091
Current	398	94	89	490	0.839041	0.182752	1.895	-0.043
Subject 2	True Negative	False Negative	False Positive	True Positive	Hit Rate	False Alarm Rate	d'	C
**Cortex**								
Previous	3747	534	653	634	0.542808	0.148409	1.151	0.468
Current	3803	198	739	828	0.807018	0.162704	1.85	0.058
**Thalamus**								
Previous	454	100	176	468	0.823944	0.279365	1.515	-0.173
Current	561	75	90	472	0.862888	0.138249	2.182	-0.003
Subject 3	True Negative	False Negative	False Positive	True Positive	Hit Rate	False Alarm Rate	d'	C
**Cortex**								
Previous	213	182	21	156	0.461538	0.089744	1.246	0.72
Current	215	58	77	222	0.792857	0.263699	1.448	-0.092
**Thalamus**								
Previous	633	65	172	123	0.654255	0.213665	1.191	0.198
Current	705	27	137	124	0.821192	0.162708	1.903	0.032

Supplementary Table 1.
Discrimination table of the two spindle detection methods. For each subject, the calculations were performed over 10 minutes of N2 and 10 minutes of N3 that had been manually marked for spindles. Cortical and thalamic channels are considered separately for each subject. “Previous” refers to the method adapted from (*1*), which is described in the Methods. “Current” refers to our current spindle detection method. The Hit Rate was calculated by dividing the number of True Positives by the sum of the True Positives and False Negatives. The False Alarm Rate was calculated by dividing the number of False Positives by the sum of False Positives and True Negatives. True negatives in the current method were candidate epochs that were not automatically selected as spindles and were not manually marked as spindles. In order to approximate the number of true negatives in the previous method, the sum of the False Negatives, False Positives, and True Positives for the previous method was subtracted from the sum of the True Negatives, False Negatives, False Positives, and True Positives separately for the cortex and thalamus for each subject. The d’, which measures discriminability, and C, which measures bias, were calculated using a d’ calculator (http://memory.psych.mun.ca/models/dprime/).

A. Cortical channels							
Subject	Channel Location	Downstate Density (/min) Overall	Spindle Density (/min) Overall	Downstate Density (/min) N2	Spindle Density (/min) N2	Downstate Density (/min) N3	Spindle Density (/min) N3
Subject 1	Middle Frontal Gyrus (1)	17.63	4.80	11.10	3.09	29.39	7.87
	Middle Frontal Gyrus (2)	17.11	6.89	9.52	4.29	30.79	11.58
	Middle Frontal Gyrus (3)	17.08	4.65	9.82	4.66	30.17	4.63
	Post Central (1)	16.49	4.17	9.15	1.15	29.72	9.62
	Post Central (2)	15.83	3.03	10.86	2.93	24.78	3.21
	Posterior Cingulate	17.31	4.92	10.64	2.39	29.34	9.49
	Angular Gyrus	16.19	4.41	8.53	2.89	29.99	7.16
Subject 2	Middle Frontal Gyrus	15.87	6.13	8.36	4.58	25.65	8.14
	Gyrus Rectus	15.11	2.68	8.56	1.81	23.64	3.80
	Orbital Gyrus	17.27	4.28	11.08	2.69	25.33	6.36
	Inferior Frontal Gyrus, Pars Orbitalis	16.40	4.98	10.59	3.61	23.97	6.77
	Inferior Frontal Gyrus (1)	16.74	3.85	9.34	1.75	26.39	6.58
	Inferior Frontal Gyrus (2)	18.06	2.96	10.67	1.19	27.68	5.27
	Frontal Operculum	15.93	3.62	9.09	0.91	24.85	7.14
	Supplementary Motor Area (1)	16.14	6.19	9.10	3.97	25.33	9.08
	Supplementary Motor Area (2)	16.29	4.88	9.59	3.30	25.03	6.94
	Supplementary Motor Area/F1	16.05	6.62	9.67	4.05	24.37	9.97
	Posterior Insula	16.71	6.19	9.84	5.28	25.66	7.37
	Precuneus	16.40	4.70	10.31	2.91	24.33	7.05
	Superior Parietal Lobule	16.06	7.24	9.28	5.50	24.89	9.51
Subject 3	Posterior Part of Supramarginal Gyrus	14.58	4.89	11.26	3.69	20.36	6.96
	Annectant Gyrus	13.11	6.77	12.27	3.50	14.58	12.44
	Averages ± SD	16.29 ± 1.06	4.95 ± 1.33	9.94 ± 1.02	3.19 ± 1.30	25.74 ± 3.64	7.59 ± 2.30
**B. Thalamic Channels**							
Subject	Channel Location	Downstate Density (/min)	Spindle Density (/min)	Downstate Density (/min) N2	Spindle Density (/min) N2	Downstate Density (/min) N3	Spindle Density (/min) N3
Subject 1	Medial Pulvinar (1)	12.22	5.88	9.39	3.90	17.30	9.46
	Medial Pulvinar (2)	11.93	10.40	9.66	7.68	16.03	15.30
	Medial Pulvinar (3)	13.22	8.45	11.80	6.38	15.79	12.18
Subject 2	Central Lateral	10.75	6.29	9.78	3.52	12.02	9.89
	Central Lateral/ Medial Pulvinar	13.34	8.40	13.08	4.97	13.68	12.88
	Medial Pulvinar/ Lateral Posterior	13.49	6.73	14.09	4.59	12.72	9.52
Subject 3	Medial Pulvinar/ Lateral Pulvinar	8.98	5.41	10.65	4.09	6.07	7.71
	Lateral Pulvinar/ Nucleus Reticularis	7.64	5.24	9.13	4.59	5.04	6.37
	Averages ± SD	11.45 ± 2.17	7.10 ± 1.81	10.95 ± 1.85	4.96 ± 1.40	12.33 ± 4.55	10.41 ± 2.90

Supplementary Table 2.
Downstate and spindle densities (rate of occurrence) overall (N2 and N3 combined), and separately for N2 and N3. A. Downstate and spindle densities for individual cortical channels. B. Downstate and spindle densities for individual thalamic channels. When N2 and N3 are combined, all but one of the cortical channels exhibit overall downstates at a higher density than all of the thalamic channels. Conversely, thalamic channels tend to exhibit a higher overall spindle density than cortical channels. A two-way ANOVA comparing the downstate densities and spindle densities of the 22 cortical channels and 8 thalamic channels found a significant

main effect of the sleep event type (downstate or spindle, $p<0.0001$), a significant main effect of location (cortical or thalamic, $p<0.0023$), and a significant interaction effect ($p<0.0001$) on the frequency of these sleep events. The rare cortical channels that spindle more often than some individual thalamic channels were largely located in posterior areas, with two located in the middle frontal gyrus. Downstate and spindle densities overlapped more between cortical and thalamic channels during N2. Overall, however, in N3, downstate density was 2.1 times greater in cortical channels (25.74) than thalamic channels (12.33), while spindle density was about 1.4 times greater in thalamic channels (10.41) than cortical channels (7.59). In the cortical channels, both downstate (9.94 to 25.74) and spindle (3.19 to 7.59) densities increase between N2 and N3. In the thalamic channels, downstate density only slightly increases between N2 and N3 (10.95 to 12.33), while the spindle density more than doubles (4.96 to 10.41). Transitioning from N2 to N3, therefore, does not have much effect on downstate density in the thalamus, while it more than doubles the cortical downstate density. Conversely, transitioning from N2 to N3 increases spindle density in both the thalamus and the cortex, but spindling still occurs more often in the thalamus.

A. Downstates				B. Spindles			
**A1. Cortex Leads Thalamus**				**B1. Thalamus Leads Cortex**			
Subject	Leading channel location	Following channel location	p-value	Subject	Leading channel location	Following channel location	p-value
Subject 1	Middle Frontal Gyrus (1)	Medial Pulvinar (1)	p<0.000001	Subject 1	Medial Pulvinar (1)	Middle Frontal Gyrus (1)	p<0.000001
	Middle Frontal Gyrus (2)	Medial Pulvinar (1)	p<0.000001		Medial Pulvinar (1)	Middle Frontal Gyrus (2)	p<0.000001
	Middle Frontal Gyrus (3)	Medial Pulvinar (1)	p<0.000001		Medial Pulvinar (2)	Middle Frontal Gyrus (1)	p<0.000001
	Post Central (1)	Medial Pulvinar (1)	p<0.000001		Medial Pulvinar (2)	Middle Frontal Gyrus (2)	p<0.000001
	Posterior Cingulate	Medial Pulvinar (1)	p<0.000001		Medial Pulvinar (2)	Post Central (1)	p<0.000001
	Angular Gyrus	Medial Pulvinar (1)	p<0.000001		Medial Pulvinar (2)	Angular Gyrus	p<0.000001
	Middle Frontal Gyrus (1)	Medial Pulvinar (2)	p<0.000001		Medial Pulvinar (3)	Middle Frontal Gyrus (1)	p<0.000001
	Post Central (2)	Medial Pulvinar (2)	p<0.000001		Medial Pulvinar (3)	Middle Frontal Gyrus (2)	p<0.000001
	Middle Frontal Gyrus (1)	Medial Pulvinar (3)	p<0.000001		Medial Pulvinar (3)	Post Central (1)	p<0.000001
	Middle Frontal Gyrus (2)	Medial Pulvinar (3)	p<0.000001		Medial Pulvinar (3)	Angular Gyrus	0.00002
	Middle Frontal Gyrus (3)	Medial Pulvinar (3)	p<0.000001				
	Post Central (1)	Medial Pulvinar (3)	p<0.000001				
	Posterior Cingulate	Medial Pulvinar (3)	0.000008				
	Angular Gyrus	Medial Pulvinar (3)	p<0.000001				
Subject 2	Middle Frontal Gyrus	Central Lateral	p<0.000001	Subject 2	Central Lateral	Middle Frontal Gyrus	p<0.000001
	Gyrus Rectus	Central Lateral	0.00001		Central Lateral	Orbital Gyrus	p<0.000001
	Orbital Gyrus	Central Lateral	0.000182		Central Lateral	Pars Orbitalis	p<0.000001
	Pars Orbitalis	Central Lateral	0.000028		Central Lateral	Area (1)	0.000066
	Middle Frontal Gyrus	Central Lateral/ Medial Pulvinar	p<0.000001		Central Lateral	Supplementary Motor Area (2)	0.000083
	Supplementary Motor Area (1)	Central Lateral/ Medial Pulvinar	p<0.000001		Central Lateral/ Medial Pulvinar	Middle Frontal Gyrus	p<0.000001
	Posterior Insula	Central Lateral/ Medial Pulvinar	p<0.000001		Central Lateral/ Medial Pulvinar	Inferior Frontal Gyrus, Pars Orbitalis	0.000057
	Precuneus	Central Lateral/ Medial Pulvinar	0.000048		Central Lateral/ Medial Pulvinar	Supplementary Motor Area (1)	p<0.000001
	Middle Frontal Gyrus	Medial Pulvinar/ Lateral Posterior	0.000003		Central Lateral/ Medial Pulvinar	Supplementary Motor Area (2)	p<0.000001
	Supplementary Motor Area (1)	Medial Pulvinar/ Lateral Posterior	0.000001		Central Lateral/ Medial Pulvinar	Supplementary Motor Area/F1	p<0.000001
	Posterior Insula	Medial Pulvinar/ Lateral Posterior	0.000044		Central Lateral/ Medial Pulvinar	Superior Parietal Lobule	0.000242
Subject 3	Posterior Part of Supramar	Medial Pulvinar/ Lateral Pulvinar	p<0.000001		Medial Pulvinar/ Lateral Posterior	Middle Frontal Gyrus	p<0.000001
	Annectant Gyrus	Medial Pulvinar/ Lateral Pulvinar	p<0.000001		Medial Pulvinar/ Lateral Posterior	Orbital Gyrus	0.000117
	Annectant Gyrus	Lateral Pulvinar/ Nucleus Reticularis	p<0.000001		Medial Pulvinar/ Lateral Posterior	Inferior Frontal Gyrus, Pars Orbitalis	0.000002
				Subject 3	Medial Pulvinar/ Lateral Pulvinar	Posterior Part of Supramar	p<0.000001
					Medial Pulvinar/ Lateral Pulvinar	Annectant Gyrus	p<0.000001
					Lateral Pulvinar/ Nucleus Reticularis	Annectant Gyrus	p<0.000001
**A2. Thalamus Leads Cortex**				**B2. Cortex Leads Thalamus**			
Subject	Leading channel location	Following channel location	p-value	Subject	Leading channel location	Following channel location	p-value
Subject 1	Medial Pulvinar (2)	Middle Frontal Gyrus (3)	0.00001	None			
Subject 2	Central Lateral	Frontal Operculum	p<0.000001				
	Central Lateral	Supplementary Motor Area/F1	p<0.000001				
	Central Lateral	Posterior Insula	0.0005				

Supplementary Table 3.
Significant binomial tests over all subjects for downstate peaks and spindle starts between all thalamocortical pairs. Bonferroni correction at $p<0.05$ was applied over all thalamocortical pairs over all subjects, separately for downstates and spindles.

**A. N2 Downstates**				**B. N2 Spindles**			
**A1. Cortex Leads Thalamus**				**B1. Thalamus Leads Cortex**			
Subject	Leading channel	Following channel	p-value	Subject	Leading channel	Following channel	p-value
Subject 1	Middle Frontal Gyrus (1)	Medial Pulvinar (1)	p<0.000001	Subject 1	Medial Pulvinar (1)	Middle Frontal Gyrus (1)	0.000534
	Middle Frontal Gyrus (2)	Medial Pulvinar (1)	p<0.000001		Medial Pulvinar (2)	Middle Frontal Gyrus (1)	p<0.000001
	Middle Frontal Gyrus (3)	Medial Pulvinar (1)	p<0.000001		Medial Pulvinar (2)	Middle Frontal Gyrus (2)	p<0.000001
	Post Central (1)	Medial Pulvinar (1)	3.50E-05		Medial Pulvinar (2)	Post Central (1)	1.00E-04
	Posterior Cingulate	Medial Pulvinar (1)	1.20E-05		Medial Pulvinar (2)	Angular Gyrus	p<0.000001
	Angular Gyrus	Medial Pulvinar (1)	p<0.000001		Medial Pulvinar (3)	Middle Frontal Gyrus (1)	p<0.000001
	Middle Frontal Gyrus (1)	Medial Pulvinar (2)	p<0.000001		Medial Pulvinar (3)	Middle Frontal Gyrus (2)	p<0.000001
	Post Central (2)	Medial Pulvinar (2)	p<0.000001		Medial Pulvinar (3)	Middle Frontal Gyrus (3)	0.000257
	Middle Frontal Gyrus (1)	Medial Pulvinar (3)	p<0.000001		Medial Pulvinar (3)	Post Central (1)	1.00E-04
	Middle Frontal Gyrus (2)	Medial Pulvinar (3)	p<0.000001	Subject 2	Central Lateral	Middle Frontal Gyrus	p<0.000001
	Middle Frontal Gyrus (3)	Medial Pulvinar (3)	3.00E-06		Central Lateral	Orbital Gyrus	p<0.000001
	Post Central (1)	Medial Pulvinar (3)	p<0.000001		Central Lateral	Inferior Frontal Gyrus, Pars Orbitalis	p<0.000001
	Angular Gyrus	Medial Pulvinar (3)	0.000219		Central Lateral/ Medial Pulvinar	Middle Frontal Gyrus	p<0.000001
Subject 2	Middle Frontal Gyrus	Central Lateral	1.50E-05		Central Lateral/ Medial Pulvinar	Supplementary Motor Area (1)	p<0.000001
	Middle Frontal Gyrus	Central Lateral/ Medial Pulvinar	2.00E-06		Central Lateral/ Medial Pulvinar	Supplementary Motor Area (2)	p<0.000001
	Supplementary Motor Area (1)	Central Lateral/ Medial Pulvinar	p<0.000001		Central Lateral/ Medial Pulvinar	Supplementary Motor Area/F1	0.000257
	Posterior Insula	Central Lateral/ Medial Pulvinar	p<0.000001		Medial Pulvinar/ Lateral Posterior	Middle Frontal Gyrus	p<0.000001
	Posterior Insula	Medial Pulvinar/ Lateral Posterior	3.00E-06		Medial Pulvinar/ Lateral Posterior	Orbital Gyrus	0.000337
Subject 3	Posterior Part of Supramar	Medial Pulvinar/ Lateral Pulvinar	p<0.000001		Medial Pulvinar/ Lateral Posterior	Inferior Frontal Gyrus, Pars Orbitalis	1.30E-05
	Annectant Gyrus	Medial Pulvinar/ Lateral Pulvinar	p<0.000001		Medial Pulvinar/ Lateral Posterior	Supplementary Motor Area (1)	p<0.000001
	Annectant Gyrus	Lateral Pulvinar/ Nucleus Reticularis	p<0.000001	Subject 3	Medial Pulvinar/ Lateral Pulvinar	Posterior Part of Supramarginal Gyrus	p<0.000001
					Medial Pulvinar/ Lateral Pulvinar	Annectant Gyrus	2.00E-06
**A2. Thalamus Leads Cortex**				**B2. Cortex Leads Thalamus**			
Subject	Leading channel	Following channel	p-value	Subject	Leading channel	Following channel	p-value
Subject 2	Central Lateral	Frontal Operculum	5.00E-06	Subject 1	Post Central (2)	Medial Pulvinar (1)	0.000306
	Central Lateral	Supplementary Motor Area (1)	p<0.000001				
	Central Lateral	Supplementary Motor Area (2)	p<0.000001				
	Central Lateral	Supplementary Motor Area/F1	1.00E-06				

Supplementary Table 4.

Binomial tests for downstates and spindles for N2 only. The results are similar to N3 in Supplementary Table 5, and to the combined N2 and N3 results in Supplementary Table 3. The lone exception is the significant spindle binomial pair in Subject 1, where the post central channel leads the medial pulvinar channel in N2 (highlighted in yellow), but not in N3, or N2 and N3 combined.

A. N3 Downstates				B. N3 Spindles			
**A1. Cortex Leads Thalamus**				**B1. Thalamus Leads Cortex**			
Subject	Leading channel	Following channel	p-value	Subject	Leading channel	Following channel	p-value
Subject 1	Middle Frontal Gyrus (1)	Medial Pulvinar (1)	2.00E-06	Subject 1	Medial Pulvinar (1)	Middle Frontal Gyrus (1)	p<0.000001
	Middle Frontal Gyrus (2)	Medial Pulvinar (1)	p<0.000001		Medial Pulvinar (1)	Middle Frontal Gyrus (2)	p<0.000001
	Middle Frontal Gyrus (3)	Medial Pulvinar (1)	p<0.000001		Medial Pulvinar (2)	Middle Frontal Gyrus (1)	p<0.000001
	Post Central (1)	Medial Pulvinar (1)	p<0.000001		Medial Pulvinar (2)	Middle Frontal Gyrus (2)	p<0.000001
	Posterior Cingulate	Medial Pulvinar (1)	p<0.000001		Medial Pulvinar (2)	Post Central (1)	5.00E-06
	Angular Gyrus	Medial Pulvinar (1)	p<0.000001		Medial Pulvinar (3)	Middle Frontal Gyrus (1)	p<0.000001
	Middle Frontal Gyrus (1)	Medial Pulvinar (2)	p<0.000001		Medial Pulvinar (3)	Middle Frontal Gyrus (2)	p<0.000001
	Post Central (2)	Medial Pulvinar (2)	p<0.000001		Medial Pulvinar (3)	Post Central (1)	0.000152
	Middle Frontal Gyrus (1)	Medial Pulvinar (3)	p<0.000001	Subject 2	Central Lateral	Middle Frontal Gyrus	p<0.000001
	Middle Frontal Gyrus (2)	Medial Pulvinar (3)	p<0.000001		Central Lateral	Orbital Gyrus	5.90E-05
	Middle Frontal Gyrus (3)	Medial Pulvinar (3)	p<0.000001		Central Lateral	Inferior Frontal Gyrus, Pars Orbitalis	p<0.000001
	Post Central (1)	Medial Pulvinar (3)	p<0.000001		Central Lateral	Supplementary Motor Area (1)	0.000695
	Angular Gyrus	Medial Pulvinar (3)	2.30E-05		Central Lateral	Supplementary Motor Area (2)	0.000503
Subject 2	Middle Frontal Gyrus	Central Lateral	p<0.000001		Central Lateral/ Medial Pulvinar	Middle Frontal Gyrus	p<0.000001
	Gyrus Rectus	Central Lateral	p<0.000001		Central Lateral/ Medial Pulvinar	O'10-11	0.000673
	Orbital Gyrus	Central Lateral	0.000253		Central Lateral/ Medial Pulvinar	Supplementary Motor Area (1)	p<0.000001
	Inferior Frontal Gyrus, Pars Orbitalis	Central Lateral	5.00E-06		Central Lateral/ Medial Pulvinar	Supplementary Motor Area (2)	p<0.000001
	Middle Frontal Gyrus	Central Lateral/ Medial Pulvinar	p<0.000001		Central Lateral/ Medial Pulvinar	Supplementary Motor Area/F1	2.00E-06
	Supplementary Motor Area (1)	Central Lateral/ Medial Pulvinar	p<0.000001		Medial Pulvinar/ Lateral Posterior	Middle Frontal Gyrus	p<0.000001
	Posterior Insula	Central Lateral/ Medial Pulvinar	p<0.000001	Subject 3	Medial Pulvinar/ Lateral Pulvinar	Posterior Part of Supramarginal Gyrus	p<0.000001
	Precuneus	Central Lateral/ Medial Pulvinar	3.00E-06		Medial Pulvinar/ Lateral Pulvinar	Annectant Gyrus	p<0.000001
	Superior Parietal Lobule	Central Lateral/ Medial Pulvinar	2.10E-05		Lateral Pulvinar/ Nucleus Reticularis	Annectant Gyrus	p<0.000001
	Middle Frontal Gyrus	Medial Pulvinar/ Lateral Posterior	0.000227				
	Supplementary Motor Area (1)	Medial Pulvinar/ Lateral Posterior	1.90E-05				
Subject 3	Annectant Gyrus	Lateral Pulvinar/ Nucleus Reticularis	7.50E-05				
**A2. Thalamus Leads Cortex**				**B2. Cortex Leads Thalamus**			
Subject	Leading channel	Following channel	p-value	Subject	Leading channel	Following channel	p-value
Subject 1	Medial Pulvinar (2)	Inferior Frontal Gyrus (2)	0.000561	None			
Subject 2	Central Lateral	Frontal Operculum	9.50E-05				
	Central Lateral	Supplementary Motor Area/F1	0.000155				

Supplementary Table 5.
Binomial tests for downstates and spindles for N3 only. The results are similar to N2 in Supplementary Table 4, and to the combined N2 and N3 results in Supplementary Table 3.

A. Cortical channels				
**Subject**	**Channel Location**	**Proportion of Spindles with Downstates**	**Normalized Proportion of Spindles with Downstates**	**Enrichment Factor**
Subject 1	Middle Frontal Gyrus	0.38	0.38	2.46
	Middle Frontal Gyrus	0.40	0.42	2.87
	Middle Frontal Gyrus	0.21	0.21	1.35
	Post Central	0.38	0.41	3.28
	Post Central	0.32	0.36	2.82
	Posterior Cingulate	0.45	0.46	5.29
	Angular Gyrus	0.32	0.35	3.39
Subject 2	Middle Frontal Gyrus	0.28	0.32	2.11
	Gyrus Rectus	0.26	0.31	2.15
	Orbital Gyrus	0.33	0.35	3.04
	Inferior Frontal Gyrus, Pars Orbitalis	0.29	0.32	2.66
	Inferior Frontal Gyrus	0.37	0.4	3.19
	Inferior Frontal Gyrus	0.46	0.46	4.43
	Frontal Operculum	0.41	0.46	3.01
	Supplementary Motor Area	0.37	0.41	3.28
	Supplementary Motor Area	0.43	0.47	4.37
	Supplementary Motor Area/F1	0.41	0.46	3.79
	Posterior Insula	0.39	0.42	3.51
	Precuneus	0.36	0.4	3.63
	Superior Parietal Lobule	0.37	0.42	3.90
Subject 3	Posterior Part of Supramarginal Gyrus	0.23	0.23	1.94
	Annectant Gyrus	0.24	0.27	2.91
	Averages ± SD	0.35 ± 0.07	0.38 ± 0.07	3.15 ± 0.9

B. Thalamic channels				
**Subject**	**Channel Location**	**Proportion of Spindles with Downstates**	**Normalized Proportion of Spindles with Downstates**	**Enrichment Factor**
Subject 1	Medial Pulvinar	0.39	0.56	4.63
	Medial Pulvinar	0.39	0.57	5.90
	Medial Pulvinar	0.56	0.74	5.80
Subject 2	Central Lateral	0.42	0.70	5.26
	Central Lateral/ Medial Pulvinar	0.69	0.94	9.86
	Medial Pulvinar/ Lateral Posterior	0.60	0.80	6.43
Sbuject 3	Medial Pulvinar/ Lateral Pulvinar	0.30	0.49	4.82
	Lateral Pulvinar/ Nucleus Reticularis	0.14	0.26	2.25
	Averages ± SD	0.43 ± 0.18	0.63 ± 0.21	5.62 ± 2.13

Supplementary Table 6.
Downstates strongly modulate spindles in the thalamus compared to the cortex. The number of spindles occurring with downstates was calculated for each channel. Due to their different relationship to downstate peaks, slightly different timing criteria were used for cortical (A) and

thalamic (B) channels: 0-750ms around the downstate peak for cortical channels and -500ms to + 250ms around the downstate peak for thalamic channels. The proportion of spindles occurring with downstates was then calculated by dividing the number of spindles that occurred within these specified time windows divided by the total number of spindles. The thalamic channels exhibited a higher proportion of downstate-related spindles (0.43 ± 0.18) compared to cortical channels (0.35 ± 0.07). When these proportions were normalized to account for the larger number of downstates occurring in the cortex (see Methods), the normalized proportion of spindles occurring with downstates is even larger between thalamic channels (0.63 ± 0.21) and cortical channels (0.38 ± 0.07). The enrichment factor measures how spindle density increases in a channel when spindles occur in relation to downstates compared to the channel's overall spindle density (see Methods). The thalamic channels show greater enrichment factors (5.62 ± 2.13) compared to the cortical channels (3.15 ± 0.9), further indicating that spindles in the thalamus are more highly modulated by the downstate than in the cortex.

Supplementary Note 1. Convergence of cortical downstates leads to a thalamic downstate

The binomial results indicate a statistically significant tendency for cortical downstates to lead thalamic downstates; however, examination of connected thalamocortical pairs still finds that the number of cortical downstates leading to thalamic downstates remains low (Supplementary Fig. 1A, boxed histograms). For example, even though the posterior insula downstates strikingly cluster just prior to the central lateral/medial pulvinar downstates, only 1258 out of 5079 posterior insula downstates, or ~25%, are followed by central lateral/medial pulvinar downstates within 500ms. The annectant gyrus and medial/lateral pulvinar pair also exhibit downstates that are tightly coupled in time to one another; however, only 400, or ~15% of the 2622 downstates in the annectant gyrus are followed by downstates in the medial/lateral pulvinar. We therefore hypothesized that a convergence of cortical downstates may be required for thalamic downstates to occur. Due to limited cortical sampling, however, a complete examination of this hypothesis is also limited. For the following analyses, only cortical bipolar pairs that were separated by at least two contacts were included.

The hypothesis that convergence from multiple cortical downstates induces thalamic downstates was tested in three ways. First, the percentage of thalamic downstates without a prior cortical downstate (in any cortical channel) within 500ms was examined for each thalamic channel. It was predicted that if a subject has more cortical channels, the percentage of isolated thalamic downstates would decrease. Subject 3 only had two cortical channels and consistent with this prediction, both thalamic channels showed a high percentage of downstates without a preceding downstate in at least one cortical channel: 67% for the Medial/Lateral Pulvinar channel and 89% for the Lateral Pulvinar/Nucleus Reticularis channel. Subject 1 included six cortical channels and Subject 2 included ten cortical channels. In both subjects, the percentage of isolated thalamic downstates decreased by ~50% compared with Subject 3: 37%, 39%, and 42% for each of the three Medial Pulvinar channels in Subject 1, with a continued drop to 35% for the Central Lateral, 30% for the Central Lateral/Medial Pulvinar, and 36% for the Medial Pulvinar/Lateral Posterior channels in Subject 2. We would predict that with robust cortical sampling, thalamic downstates would not occur without preceding cortical downstates.

Second, we examined whether cortical pairs that produce downstates together are more likely to lead to thalamic downstates, compared with cortical downstates produced in only one of these cortical channels. For each pair of cortical channels, the number of downstates occurring

within 500ms of the locked channel was calculated. The probability of thalamic downstates occurring within 500ms after these paired cortical downstates was calculated for each thalamic channel; the downstate peak of the locked cortical channel was used to search for thalamic downstates. The probability of thalamic downstates in each thalamic channel occurring within 500ms of isolated cortical downstates was also calculated for each cortical channel individually. Over all thalamic channels, the mean probability of thalamic downstates occurring with cortical pairs (0.14) was significantly greater than when thalamic downstates occur with a single cortical channel (0.10) (paired t-test, p=0.011, 8 thalamic channels). A Chi-squared test was performed between the proportion of thalamic downstates relative to isolated versus paired cortical downstates. Out of 364 unique cortical /thalamic channel combinations, 54 (~15%) were significant after Bonferroni correction at p<0.05. For an example, see Fig. 1G (in the main text). Of these, one showed a greater probability of a thalamic downstate when downstates occurred on the individual cortical channel, but the remaining 53 showed a greater probability of a thalamic downstate when the cortical downstate occurred in both cortical channels. Overall, these results indicate that thalamic downstates are more likely to occur when cortical downstates occur in two related cortical channels.

The third way we tested the convergence hypothesis was by examining the probability of a thalamic downstate as a function of the number of cortical channels showing a downstate. With small and occasional exceptions, the addition of multiple channels participating in a cortical downstate further increased the probability of a following thalamic downstate, as shown in Supplementary Fig. 2. In summary, our results indicate that as a greater number of related cortical channels participate in a downstate, the more likely a downstate will follow in the thalamus. Overall, however, the percentages are relatively low. This may suggest that very widespread convergence is needed, which was not possible to observe given our limited cortical sampling. Alternatively, upstates or another ongoing process not measured here may also be contributing to the generation of thalamic downstates.

Supplementary Note 2. Corticothalamic pair connectivity

A total of 64 unique corticothalamic pairs were sampled over all three patients. Two of these pairs were located in areas previously shown to be anatomically and functionally connected (*2-4*): the medial pulvinar and the posterior insula, and the pulvinar and the annectant gyrus (intraparietal sulcus). Both of these pairs exhibited unique relationships during downstates and spindles, which are featured in Supplementary Fig. 1 (boxed histograms) and Fig. 3C-E.

Additional pairs also demonstrated differing degrees of functional specificity, supporting growing evidence that cortico-pulvinar connections may be variable even within the medial pulvinar (*2, 5*). Within the medial pulvinar of Subject 1, we find a double dissociation where one medial pulvinar channel is highly associated with a middle frontal gyrus channel and a second medial pulvinar channel is highly associated with a post central channel. This relationship is detailed in Supplementary Fig. 8.

Supplementary References

1. Y. Nir, R. J. Staba, T. Andrillon, V. V. Vyazovskiy, C. Cirelli, I. Fried, G. Tononi, Regional slow waves and spindles in human sleep. *Neuron.* **70**, 153-169 (2011).
2. M. J. Arcaro, M. A. Pinsk, S. Kastner, The Anatomical and Functional Organization of the Human Visual Pulvinar. *Journal of Neuroscience.* **35**, 9848-9871 (2015).
3. E. J. Mufson, M. M. Mesulam, Thalamic connections of the insula in the rhesus monkey and comments on the paralimbic connectivity of the medial pulvinar nucleus. *Journal of Comparative Neurology.* **227**, 109-120 (1984).
4. D. S. Rosenberg, F. Mauguiere, H. Catenoix, I. Faillenot, M. Magnin, Reciprocal thalamocortical connectivity of the medial pulvinar: a depth stimulation and evoked potential study in human brain. *Cerebral Cortex.* **19**, 1462-1473 (2009).
5. M. Thieffry, Y. Burnod, Y. Poussart, J. Calvet, Synchronous modifications in the cortical and pulvinar unit activity during slow wave sleep. *Experimental Neurology.* **55**, 327-339 (1977).
